# Binding of Multiple Rap1 Proteins Stimulates Chromosome Breakage Induction during DNA Replication

**DOI:** 10.1371/journal.pgen.1005283

**Published:** 2015-08-11

**Authors:** Greicy H. Goto, Sevil Zencir, Yukinori Hirano, Hiroo Ogi, Andreas Ivessa, Katsunori Sugimoto

**Affiliations:** 1 Department of Microbiology, Biochemistry and Molecular Genetics, New Jersey Medical School, Rutgers, The State University of New Jersey, Newark, New Jersey, United States of America; 2 Department of Cell Biology and Molecular Medicine, New Jersey Medical School, Rutgers, The State University of New Jersey, Newark, New Jersey, United States of America; Chinese Academy of Sciences, CHINA

## Abstract

Telomeres, the ends of linear eukaryotic chromosomes, have a specialized chromatin structure that provides a stable chromosomal terminus. In budding yeast Rap1 protein binds to telomeric TG repeat and negatively regulates telomere length. Here we show that binding of multiple Rap1 proteins stimulates DNA double-stranded break (DSB) induction at both telomeric and non-telomeric regions. Consistent with the role of DSB induction, Rap1 stimulates nearby recombination events in a dosage-dependent manner. Rap1 recruits Rif1 and Rif2 to telomeres, but neither Rif1 nor Rif2 is required for DSB induction. Rap1-mediated DSB induction involves replication fork progression but inactivation of checkpoint kinase Mec1 does not affect DSB induction. Rap1 tethering shortens artificially elongated telomeres in parallel with telomerase inhibition, and this telomere shortening does not require homologous recombination. These results suggest that Rap1 contributes to telomere homeostasis by promoting chromosome breakage.

## Author Summary

Telomere length is maintained primarily through equilibrium between telomerase-mediated lengthening and the loss of telomeric sequence through the end-replication problem. In budding yeast Rap1 protein binds to telomeric TG repeat and negatively regulates telomerase recruitment in a dosage-dependent manner. In this paper we provide evidence suggesting an alternative Rap1-dependent telomere shortening mechanism in which binding of multiple Rap1 proteins mediates DNA break induction during DNA replication. This process does not involve recombination events; therefore, it is distinct from loop-mediated telomere trimming.

## Introduction

Telomeres are specialized nucleoprotein complexes at the ends of linear eukaryotic chromosomes. The DNA component of telomeres typically comprises a double-stranded DNA (dsDNA) region of a tandem repeat and a 3’ protruding single-stranded DNA (ssDNA) region of the G-rich strand [1,2]. Both the dsDNA and ssDNA regions are covered with sequence-specific binding proteins. Telomeres protect chromosome ends from degradation or fusion [2,3]. Telomeres also promote DNA replication at the chromosome ends. Since conventional DNA polymerases cannot complete DNA synthesis at telomeres, linear chromosomes shorten progressively with every round of cell division. In most eukaryotes, continuous telomere shortening can be counteracted by telomerase [4].

The length of the duplex telomeric repeat is kept within a relatively narrow range in a cell-type specific manner [1]. In cells that express telomerase, telomere length homeostasis results from a balance between telomerase-dependent telomere addition and telomere shortening. The average telomere length varies between 5 and 15 kb in human, whereas much shorter telomeres (~300 bp) are maintained in the budding yeast *Saccharomyces cerevisiae*. Telomere shortening results from incomplete DNA end replication and 5’-resection (end-replication problem) [2,5]. For example, in the absence of telomerase, telomeres of human and budding yeast cells lose 50–200 and 3–5 nucleotides per round of DNA replication, respectively [6–9]. Telomere shortening can also occur in a more accelerated manner called telomeric rapid deletion (TRD), some of which involves intrachromosomal recombination events between telomeric repeats [10–13].

The structure of telomeres in budding yeast is typical of most eukaryotes, except that the repeat unit (abbreviated TG_1-3_) is heterogeneous [2]. As in other eukaryotes, telomeres consist of double-stranded and single-stranded units in budding yeast. The G-rich strand extends to form a single-strand tail, which is covered with Cdc13 in complex with Stn1 and Ten1 [14–16]. The Cdc13-Stn1-Ten1 complex acts as a telomere cap to protect telomeres from degradation [2]. Telomerase comprises the catalytic subunit Est2, two accessary subunits (Est1 and Est3) and the RNA component Tlc1 in budding yeast [2,4]. Cdc13 interacts with Est1 and contributes to telomerase recruitment to chromosome ends [2,4]. Double-stranded telomeric DNA repeats are bound by the sequence-specific binding protein Rap1.

The budding yeast Rap1 protein is an essential protein involved in many diverse processes including transcription and telomere maintenance [17]. Rap1 consists of three conserved domains: a BRCT domain in the N-terminal region, a centrally located DNA-binding domain with two Myb-like folds, a transcription activation (TA) domain, and an independent C-terminal domain called RCT (Rap1 C-terminal) [17]. The central region, containing two Myb domains and the TA domain, plays an essential role in cell viability by regulating transcriptional activation of various genes [18]. The N-terminus has been shown to potentiate DNA bending of the Rap1-DNA complex [19]. The RCT domain regulates localization of Sir3 and Sir4 and promotes transcriptional silencing [20]. This domain also forms a complex with and recruits Rif1 and Rif2 to telomeres [21–24]. Targeting of the RCT, Rif1 or Rif2 to a telomere induces telomere shortening to an extent that is proportional to the number of targeted molecules [25,26]. This observation has led to the “protein counting model” of telomere length control, in which increasing numbers of telomere-bound Rap1 molecules negatively regulates telomerase-mediated telomere extension. The “protein counting model” has been defined by recent studies. As in other eukaryotes, the checkpoint protein kinases Mec1 and Tel1 control telomere length in budding yeast [2]. Although Mec1 is a central checkpoint kinase in the response to DNA replication block and DNA damage [27], it has only minor functions in telomere homeostasis. In contrast, Tel1 plays a major role in telomere length maintenance in budding yeast [28]. Tel1 localizes to short telomeres as well as DNA double-strand breaks (DSBs) [29–32]. In turn, Tel1 recruits telomerase to short telomeres and promotes telomere addition [32–34]. Telomere extension increases Rap1 binding sites at telomeres. Rap1 collaborates with Rif1 and Rif2 and inhibits localization of Tel1 to DNA ends [35,36]. Thus, Rap1 cooperates with Rif1 and Rif2 to impede telomerase recruitment, therefore negatively regulating telomere length.

The G-rich strand of telomeric TG repeats can fold into G-quadruplex (G4) structures, which could cause replication fork blocks and induce DNA breaks [37]. Indeed, internal tracts of TG repeats are unstable and spontaneously converted to telomeres [38]. Rap1 binds internal tracts of TG repeat sequence as well as telomeres [21,22,35]. Since Rap1 binds to duplex DNA, binding of Rap1 to TG repeat sequence could inhibit G4 structure formation. However, there is evidence suggesting that Rap1 also binds to single-stranded DNA and stimulate G4 formation [39]. Alternatively, it is formally possible that DNA binding of Rap1 promotes DNA break induction independently of the G4 structure.

In this study we provide evidence supporting that binding of multiple Rap1 proteins is involved in chromosome breakage during S phase. We devised systems to determine whether DNA binding of Rap1 stimulates DSB induction. We found that Rap1 mediates DSB induction at both non-telomeric and telomeric regions. While Rap1 operates together with Rif1 and Rif2 to inhibit telomerase recruitment, Rap1 acts independently of Rif1 or Rif2 function to induce chromosome breakage. Rap1 tethering prompts DSB generation in a copy number-dependent manner during DNA replication. While Rap1-mediated DSB induction shortens artificially extended telomeres, this telomere shortening does not involve homologous recombination events. Our results suggest that Rap1 contributes to telomere homeostasis by inducing DNA breaks.

## Results

### Rap1-dependent chromosome truncation at TG repeats

**Fig 1:**
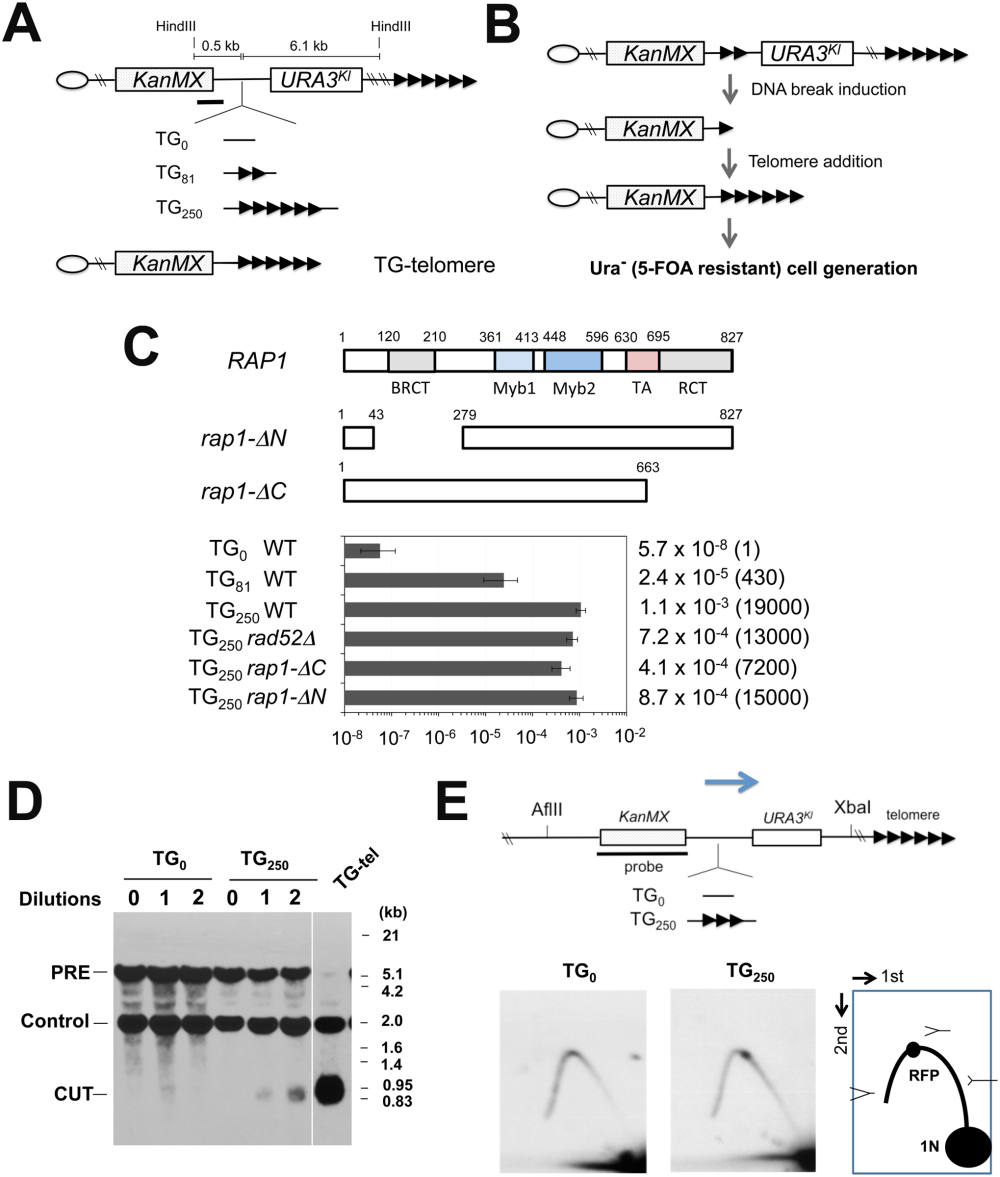
Chromosome truncation at internal TG repeats. (A) Schematic of the 81 bp TG (TG_81_) or 250 bp TG (TG_250_) repeat at the *ADH4* locus on chromosome VII-L. The *ADH4* locus was replaced with a DNA fragment containing the *KanMX* gene and the *URA3* ^*Kl*^ gene. The TG_81_ or TG_250_ sequence was introduced between *KanMX* and *URA3* ^*Kl*^. The cassette with no TG sequence was called as TG_0_. The centromere is shown as a circle on the left (CEN) and the telomere is shown as repetitive arrows on the right. The black line indicates a hybridization probe for Southern blot (Fig 1C). TG-telomere cells contain a telomere at the position where the TG_250_ repeat is inserted (See S1 Fig for more details). (B) Schematic of experimental protocol to detect *URA3* ^*Kl*^ marker loss after telomere addition at the TG repeat sequences. (C) Effect of TG_81_ or TG_250_ insertion on *URA3* marker loss. Cells containing the TG_0_, TG_81_ or TG_250_ cassette were first maintained in medium selective for *URA3* and then transferred to non-selective medium. Saturated cultures were diluted and spread out on 5-FOA plates to determine the rate of *URA3* ^*Kl*^ marker loss. *URA3* ^*Kl*^ marker loss rate per generation was quantified through fluctuation analysis. Error bars represent 95% confidence intervals. Number in parentheses indicates rate relative to cells containing the TG_0_ cassette. Rap1 contains a BRCT domain, two Myb domains, a transcription activation (TA) domain and a C-terminal RCT domain. The *rap1-ΔN* and the *rap1-ΔC* mutation lack the BRCT and the RCT domain, respectively. (D) Effect of TG_250_ insertion on chromosome truncation. Cells containing the TG_0_ or TG_250_ cassette were first maintained in medium selective for *URA3* and then diluted 1000-fold in non-selective medium to allow cells to grow for one day (10 generations) and aliquots were collected for genomic DNA preparation. This cycle was repeated again. DNA was digested with HindIII and analyzed by Southern blot using the probe shown in A. DNA breakage at TG_250_ generates a 0.7 kb fragment (marked with CUT) from the 6.9 kb fragment (PRE). The probe also detects a 1.8 kb HindIII fragment (Control) from the *SMC2* locus on chromosome VI. TG-telomere (TG-tel) serves as a control to detect DSB induction at the TG_250_ sequence. (E) Effect of TG_250_ insertion on replication fork pausing. Cells containing the TG_0_ or TG_250_ cassette were first maintained in medium selective for *URA3* and then transferred to non-selective medium to allow cells to undergo cell division for 4 hr. CsCl gradient purified DNA was digested with AflII and XbaI and analyzed by two-dimensional gel electrophoresis using the indicated probe. The probe detects a 6.3 kb AflII-XbaI fragment. The TG_250_ repeat locates 3.0 kb from the AflII site and 2.9 kb from the XbaI site. RFP represents replication fork pausing. The arrow indicates the direction of replication fork movement. There is a highly active replication origin 40 kb proximal to the repeat insert site (the *ADH4* locus) on chromosome VII [82].

We examined whether Rap1 is involved in DSB induction, thereby converting internal tracts of TG sequence to telomeres. To this end, we placed an 81 bp (TG_81_) or a 250 bp telomeric TG (TG_250_) repeat sequence between the *KanMX* marker and the *K*. *lactis URA3* ^*Kl*^ gene at the *ADH4* locus (Fig 1A). The TG_81_ or TG_250_ sequence, derived from endogenous telomeres, contains four or twelve Rap1 binding motifs, respectively [36,40]. There is no essential gene from the *ADH4* locus to the chromosome end. Eukaryotes utilize two major pathways for DSB repair; homologous recombination (HR) and non-homologous end joining (NHEJ) [27]. In budding yeast, HR is the central DSB repair pathway. However, HR cannot efficiently repair centromere-proximal DSB ends generated between *KanMX* and *URA3* ^*Kl*^ because there is no homologous donor sequence available (see below). Instead, telomerase-dependent telomere addition occurs at DNA ends with telomeric TG repeat sequence nearby [25,40], a phenomenon which is referred to as “telomere healing” (Fig 1B). Although *URA3* cells cannot proliferate on medium containing 5-fluoroorotic acid (5-FOA), *ura3* mutant cells can [41]. Therefore, if a DNA break is induced within or near the TG sequence, telomere formation can eliminate the distal portion including the *URA3* ^*Kl*^ marker gene, therefore generating 5-FOA resistant colonies. Cells were first maintained in medium selective for *URA3* cells and then transferred to non-selective medium. Saturated cultures were diluted and spread on 5-FOA plates to monitor the rate of *URA3* ^*Kl*^ marker loss (Fig 1C). The *URA3* ^*Kl*^ marker was stably maintained if there was no TG repeat (TG_0_) sequence. Introduction of the TG_250_ repeat sequence, however, stimulated loss of the *URA3* ^*Kl*^ marker very efficiently (19,000-fold). Placement of the TG_81_ repeat increased *URA3* ^*Kl*^ marker loss (430-fold) but much less efficiently compared with the TG_250_ sequence. All of the twenty Ura^-^ cells examined possessed a telomere near the TG_81_ or TG_250_ sequence (S1 Fig)[38]. Consistent with telomerase-dependent telomere addition at TG sequences, inactivation of the *RAD52*-dependent HR pathway did not affect *URA3* ^*Kl*^ marker loss [40] (Fig 1C). We examined whether chromosome breakage occurs near the TG_250_ repeat sequence by Southern blotting analysis. Cells carrying the TG_0_ or TG_250_ cassette were first cultured in medium selective for *URA3* cells and then transferred to non-selective medium. Introduction of the TG_250_ repeat accumulated cells containing a DNA end nearby (Fig 1D). Several lines of evidence have established the model in which persistent DNA fork stalling leads eventually to DSB induction [42]. Replication forks slow during their passage through telomeric TG tracts [43]. We confirmed that replication forks paused at the TG_250_ repeat sequence by two-dimensional gel electrophoresis analysis (Fig 1E and S2 Fig).

**Fig 2:**
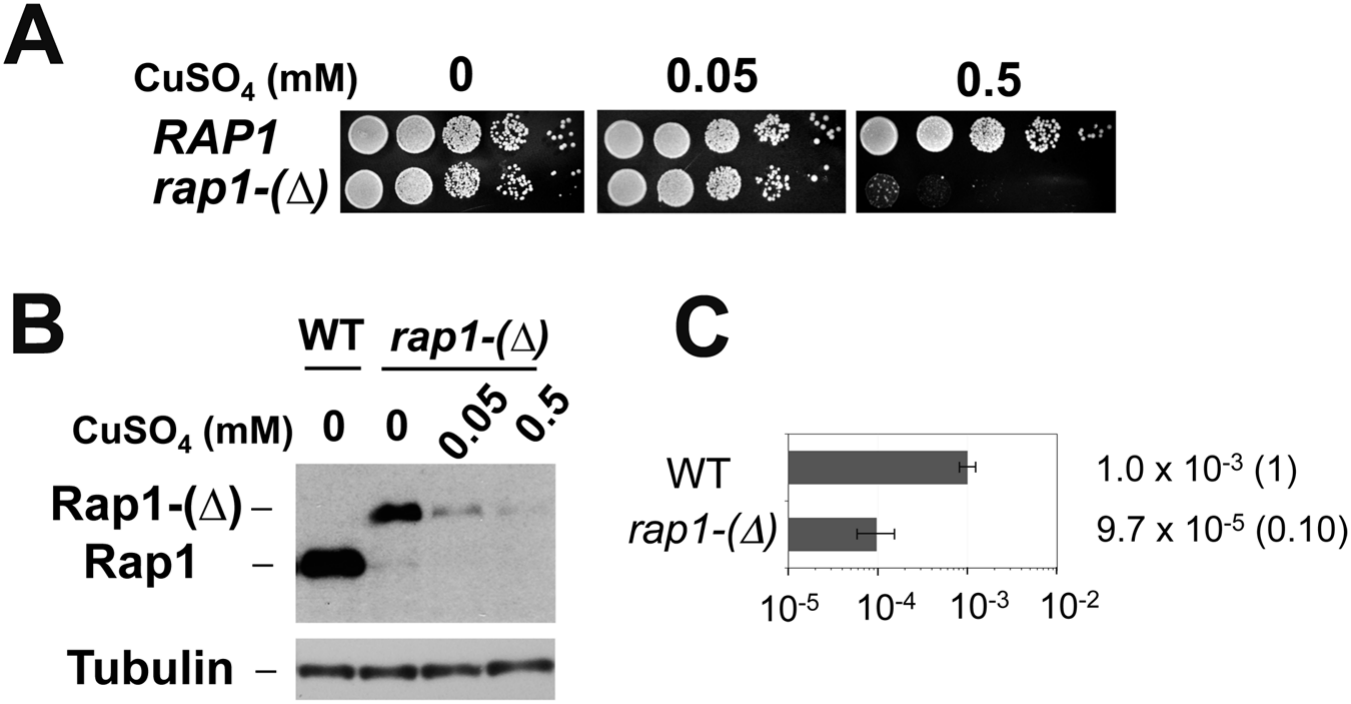
Requirement of Rap1 on chromosome truncation at TG repeats. (A) Effect of *rap1*-degron mutation on colony formation in the presence of CuSO_4_. Wild-type or *rap1*-degron mutant (*rap1-(Δ)*) cells were serially diluted (10-fold) and spotted on medium containing 0, 0.05 or 0.5 mM CuSO_4_. (B) Effect of CuSO_4_ concentration on Rap1-(Δ) protein expression. Wild-type or *rap1-(Δ)* cells were initially grown in the absence of CuSO_4_ and then incubated with the indicated concentrations of CuSO_4_ for 6 hr. Aliquots of cells were collected and subjected to immunoblotting analysis with anti-Rap1 antibodies. (C) Effect of Rap1 depletion on *URA3* ^*Kl*^ marker loss. Wild-type or *rap1-(Δ)* cells containing the TG_250_ cassette were first maintained in medium selective for *URA3* and then transferred to non-selective medium containing 0.05 mM CuSO_4_. Saturated cultures were diluted and spread on 5-FOA plates to determine the rate of *URA3* ^*Kl*^ marker loss. *URA3* ^*Kl*^ maker loss was determined as in Fig 1C.

We investigated whether Rap1 is required for *URA3* ^*Kl*^ marker loss. Since the *RAP1* gene is essential for cell proliferation, we examined the effect of partial Rap1 depletion using a copper-inducible *rap1* degron, *rap1-(Δ)* [44]. Consistent with an essential role of Rap1 in cell proliferation, *rap1-(Δ)* mutants grew very poorly in the presence of 0.5 mM CuSO_4_ (Fig 2A) as the expression of Rap1 degron protein was decreased (Fig 2B). In contrast, incubation with 0.05 mM CuSO_4_ did not significantly affect cell proliferation (Fig 2A) although the Rap1 expression level was decreased (Fig 2B). We thus investigated the effect of *rap1-(Δ)* mutation on *URA3* ^*Kl*^ marker loss in the presence of 0.05 mM CuSO_4_ (Fig 2C). Partial Rap1 depletion was found to decrease *URA3* ^*Kl*^ marker loss. These results are consistent with the hypothesis that Rap1 promotes DSB induction in a copy number-dependent manner.

Rap1 comprises three conserved domains: a BRCT domain in the N-terminal region, a central region with DNA-binding Myb and TA domains, and a C-terminal RCT domain [17] (Fig 1C). Although the central region is essential for cell viability, the BRCT or RCT domain is not [17,45,46]. To determine the role of the BRCT or the RCT domain, we examined the effect of *rap1-ΔN* or *rap1-ΔC* mutation on loss of the *URA3* ^*Kl*^ marker. However, neither the N-terminal nor the C-terminal deletion significantly affected the generation of Ura^-^ cells (Fig 1C), raising a possibility that the central region of Rap1 protein is involved in DSB induction.

### Stimulation of homologous recombination nearby by tethering of Rap1 on chromatin

**Fig 3:**
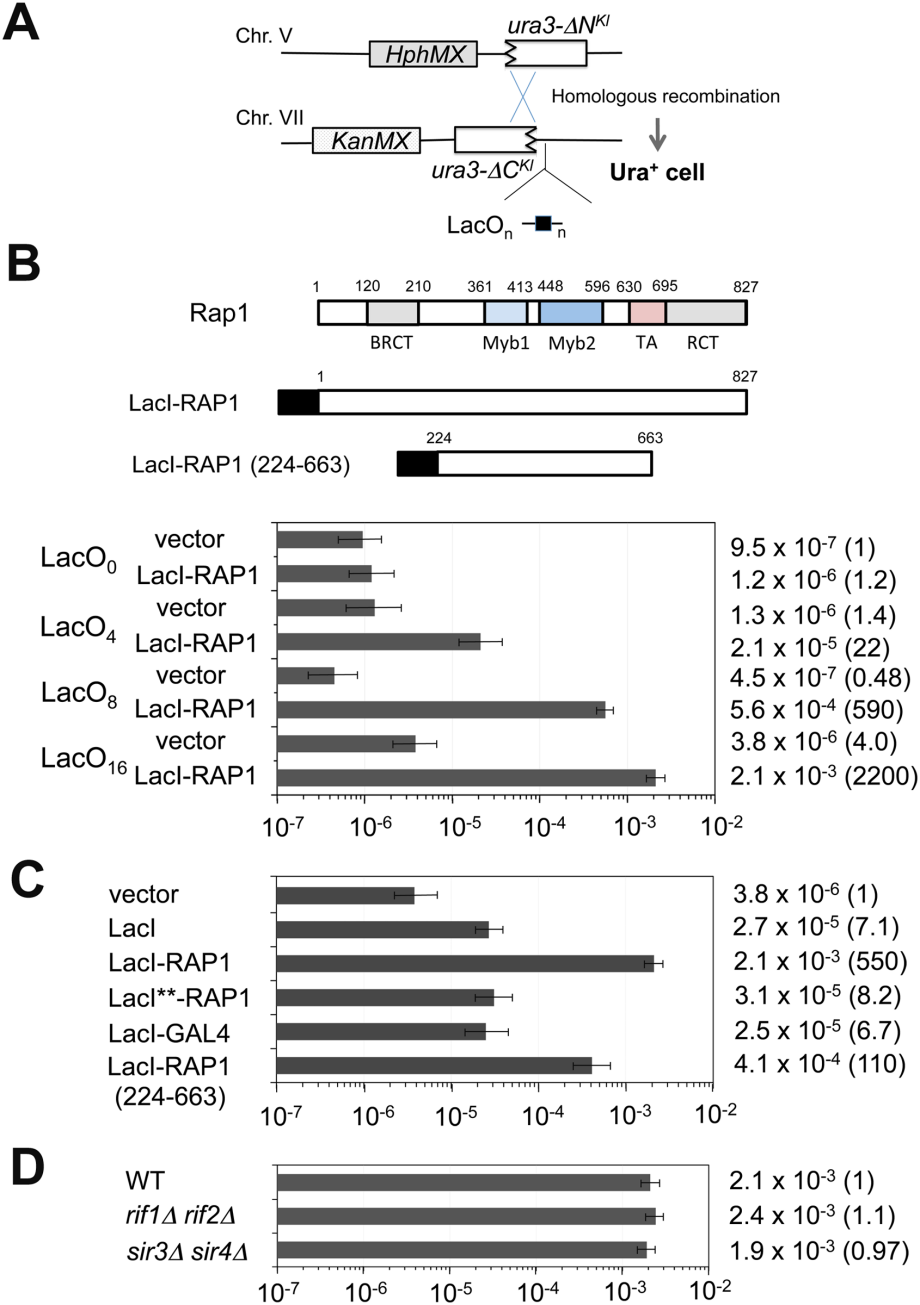
Effect of Rap1 tethering on homologous recombination between the *ura3-ΔN* ^*Kl*^ gene and the *ura3-ΔC* ^*Kl*^ gene. (A) Schematic of the ura3-ΔC-LacO cassette on chromosome VII-L and the ura3-ΔN cassette on chromosome V-R. The *YER186* locus on chromosome V was replaced with the ura3-ΔN cassette marked with the *HphMX* gene. The *ADH4* locus on chromosome VII was replaced with the *KanMX*-marked cassettes containing the *ura3-ΔC* ^*Kl*^ truncated gene and different copies (n) of the lacO sequence. There is no essential gene from the *ADH4* or *YER186* locus to the corresponding chromosome end. (B) Effect of LacI-Rap1 expression on homologous recombination near the LacO_0_, LacO_4_, LacO_8_ or LacO_16_ cassette. Fusion proteins contain the DNA-binding domain of LacI (black square). This LacI construct lacks the C-terminal oligomerization domain but contains the nuclear localization signal (PKKKRKV) derived from the SV40 Large T-antigen. Cells carrying pGAL-LacI-RAP1 or the control vector were grown in 2% sucrose and then transferred to 2% galactose and 0.5% glucose. Saturated cultures were diluted and spread on uracil dropout plates. *URA3* ^*Kl*^ homologous recombination rate per generation was determined through fluctuation analysis. Error bars represent 95% confidence intervals. Number in parentheses indicates rate relative to LacO_0_ cells carrying the control vector. (C) Effect of LacI, LacI-GAL4 or LacI-Rap1 (224–663) expression on homologous recombination near the LacO_16_ cassette. Cells carrying pGAL-LacI, pGAL-LacI-RAP1, pGAL-LacI**-RAP1, pGAL-LacI-GAL4, pGAL-LacI-RAP1 (224–663) or the control vector were cultured and examined as in (B). Number in parentheses indicates rate relative to cells containing the LacO_16_ cassette and carrying the control vector. The construction of LacI-Rap1 (224–663) is shown in (B). (D) Effect of *rif1Δ rif2Δ* or *sir3Δ sir4Δ* mutation on homologous recombination near the LacO_16_ cassette. Wild-type, *rif1Δ rif2Δ* or *sir3Δ sir4Δ* mutant cells carrying pGAL-LacI-RAP1 were cultured and examined as in (C). Number in parentheses indicates rate relative to wild-type cells containing the LacO_16_ cassette.

The above results support the model in which binding of multiple Rap1 proteins results in DSB induction. However, it remains possible that Rap1 binding mediates the formation of G-quadruplex (G4) structures at TG repeats, which could cause replication fork blocks and induce DNA breaks [37]. The TG_81_ and the TG_250_ repeat sequence can potentially form two and eight G4 structures, respectively [47]. Moreover, Rap1 binding could stimulate telomerase activity at DNA ends. To exclude these possibilities, we set up a system that recruits Rap1 to non-TG sequences (Fig 3A). We constructed a LacI-Rap1 fusion (Fig 3B), which restores proliferation to *rap1Δ* mutants (S3 Fig) and functions as a negative regulator of telomere length (see below). Expression of LacI-Rap1 fusion was driven from the *GAL1* promoter in medium containing 2% galactose and 0.5% glucose (galactose hereafter), thereby maintaining the expression level of LacI-Rap1 protein similar to that of endogenous Rap1 protein (S4 Fig). To target LacI-Rap1 to non-TG sequences, we inserted cassettes with different copy numbers of the LacI-binding sequence (lacO) (Fig 3A). No putative G4 forming sequence was found on the lacO sequences [47]. One LacI homodimer can bind to each lacO operator [48]; therefore, one lacO copy can be covered with two LacI-Rap1 molecules. However, the LacO_4_ cassette behaved like ~80 bp of telomeric TG sequence containing four Rap1 binding sites after LacI-Rap1 expression (see below). The average telomere length is ~300 bp and the Rap1 binding motif appears every 20 bp at telomeres [2]. Thus, the LacO_16_ repeat in the presence of LacI-Rap1 could correspond to wild-type length telomere (~320 bp of TG repeat sequence) in terms of Rap1 binding. We introduced a 3’-terminal truncation of *URA3* ^*Kl*^ adjacent to the lacO sequences at the *ADH4* locus and a 5’-terminal truncation of *URA3* ^*Kl*^ on a different chromosome (Fig 3A). In this system DNA breaks at the lacO sequences can be repaired by homologous recombination, generating the full-length *URA3* ^*Kl*^ marker gene. We thus determined the homologous recombination frequency between the truncated *ura3* ^*Kl*^ genes to estimate LacI-Rap1-mediated DSB induction. Cells carrying pGAL-LacI-RAP1 or the control vector were initially grown in sucrose and then incubated with galactose to express LacI-Rap1. Saturated cultures were diluted and plated to score Ura^+^ cells (Fig 3A). LacI-Rap1 expression stimulated interchromosomal recombination more efficiently when cells contained longer lacO sequences (Fig 3B). For example, LacI-Rap1 expression led to 2,000-fold higher recombination events in cells containing the LacO_16_ cassette compared to cells containing no lacO (the LacO_0_ cassette). LacI alone stimulated recombination near the LacO_16_ cassette but much less efficiently than LacI-Rap1 fusion (Fig 3C). The observed effect is not specific to LacI-fusion, since TetR-Rap1 fusion also stimulated recombination near the tetO repeat (S5 Fig).

Since the N-terminus or C-terminus of Rap1 was dispensable for TG-mediated chromosome truncation (see Fig 1C), we examined whether the central region of Rap1 stimulates interchromosomal recombination (Fig 3C). We constructed a LacI-Rap1 fusion lacking both the N- and C-termini of Rap1, named LacI-Rap1 (224–663) (Fig 3B). The LacI-Rap1 (224–663) fusion stimulated recombination strongly compared with LacI alone but weakly compared with LacI-Rap1 (Fig 3C). The expression level of LacI alone and LacI-Rap1 (224–663) was not significantly differently from that of LacI-Rap1 (S4 Fig). Although the central domain of Rap1 triggered recombination less efficiently than the full-length Rap1 protein, deletion of the central region abolished recombination stimulation; LacI-Rap1 (ΔM1-M2-TA) behaved similarly to LacI alone (S6 Fig). These results support the idea that the central region of Rap1 plays a key role in DSB induction. The central region of Rap1 consists of two Myb domains and a TA domain. Neither Myb nor TA domain was specifically involved in recombination stimulation, suggesting that the overall structure of the central region is critical for DSB induction (S6 Fig). The C-terminal region of Rap1 mediates interaction with Rif1 or Rif2 for telomere homeostasis and Sir3 or Sir4 for transcriptional repression [2,17]. In agreement with the finding that the C-terminus is dispensable for Rap1-mediated DSB induction, neither *rif1Δ rif2Δ* nor *sir3Δ sir4Δ* double mutation affected recombination (Fig 3D). It is possible that any transcriptional activation protein stimulates DSB induction similar to Rap1. Gal4 is a potent transcription activator and the calculated molecular mass of Gal4 is similar to that of Rap1. We examined the effect of LacI-Gal4 expression on DSB induction (Fig 3C). LacI-Gal4 expression increased recombination compared with the control vector but behaved like LacI alone. The expression level of LacI-Gal4 was similar to that of LacI-Rap1 (S4 Fig). Both Rap1 and LacI bind to the respective consensus sequence with a high affinity (Kd = ~1x10^-11^M) [49,50]. One explanation could be that DSB induction involves tight DNA binding. We addressed this possibility by using the LacI** variant that has weak affinity for the binding sequence but does not impair the accumulation at lacO repeat sequences [51]. We found that LacI**-Rap1 protein was defective in DSB induction (Fig 3C) although the expression of LacI-Rap1** was similar to that of LacI-Rap1 (S4 Fig). Thus, anchoring of Rap1 appears to promote DSB induction rather specifically.

### Induction of DNA breakage by tethering of Rap1 on chromatin

**Fig 4:**
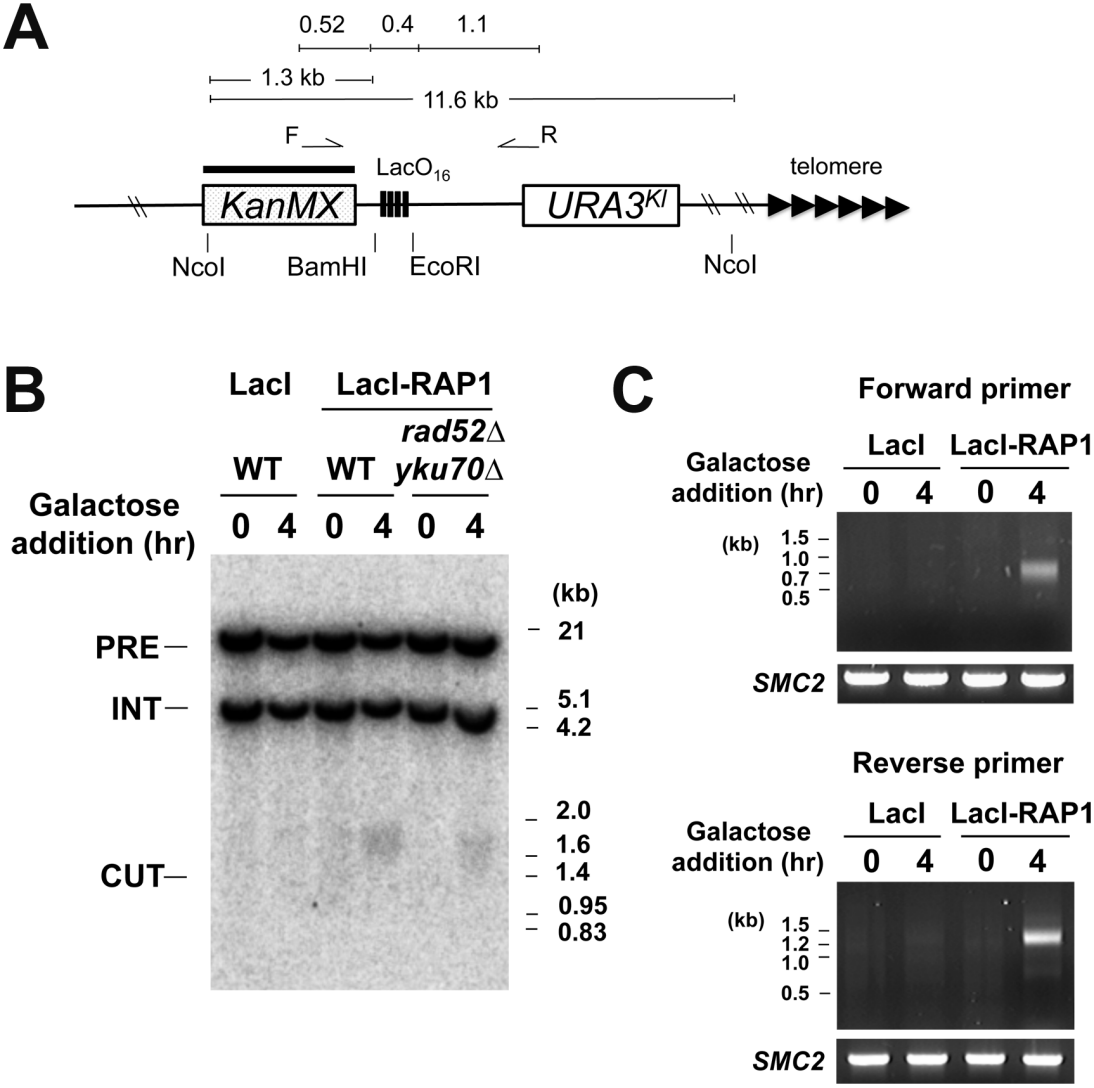
DSB induction at the LacO_16_ repeat after LacI-Rap1 expression. (A) Schematic of the LacO_16_ repeat at the *ADH4* locus on chromosome VII-L. The *ADH4* locus was marked with a DNA fragment containing the *KanMX* gene and the *URA3* ^*Kl*^ gene. The LacO_16_ sequence is inserted between the BamHI and EcoRI site. Black bar indicates a hybridization probe for Southern blot. The forward primer (F) and reverse primer (R) were used for a TdT-based DNA end detection assay. Primer F and R are 0.52 kb and 1.1 kb away from the LacO_16_ insertion site, respectively. (B) Detection of DNA breakage by Southern blot. Wild-type or *rad52Δ yku70Δ* mutant cells containing the LacO_16_-URA3 cassette were transformed with pGAL-LacI-RAP1 or pGAL-LacI. Transformants were initially grown in 2% sucrose and then incubated with 2% galactose and 0.5% glucose for 4 hr. Genomic DNA was digested with NcoI and then analyzed by Southern blot using the *KanMX* gene and a fragment from chromosome I as a probe. Hybridization detects two NcoI-fragments; one from chromosome I (5 kb, marked with INT) and the other from the chromosome VII region (11.6 kb, marked with PRE). DNA breakage at LacO_16_ generates a 1.3–1.7 kb fragment (marked with CUT) from the 11.6 kb fragment. (C) Detection of DNA ends by TdT-dependent dCTP addition. LacO_16_-URA3 cells carrying pGAL-LacI-RAP1 or pGAL-LacI were treated as in (B). Genomic DNA was incubated with TdT using dCTP as a substrate and then subjected to PCR using either the forward primer or the reverse primer with a poly(dG)-oligonucleotide. As control, the *SMC2* locus was amplified by PCR.

We investigated whether LacI-Rap1 tethering induces DNA breaks at the LacO_16_ repeat by Southern blot analysis. We used a strain carrying the LacO_16_ cassette between *KanMX* and *URA3* ^*Kl*^ at the *ADH4* locus (Fig 4A). Cells transformed with pGAL-LacI-RAP1 or pGAL-LacI were grown in sucrose and then transferred to galactose medium for 4 hr. LacI-Rap1 expression induced DNA breakage near the LacO_16_ repeat whereas no apparent cleavage was detected with LacI expression (Fig 4B). Neither Ku-dependent NHEJ nor Rad52-dependent HR significantly affected DNA breakage detection (Fig 4B). This observation is consistent with the view that NHEJ is a minor pathway in budding yeast [27] and there is no homologous donor sequence available for DNA breaks generated between *KanMX* and *URA3* ^*Kl*^. It was estimated that 3% of cells received a DNA break at the LacO_16_ locus 4 hr after LacI-Rap1 expression (S7 Fig).

The terminal deoxynucleotidyl transferase (TdT) has been used to end-label DNA ends including DSBs and telomeres [52]. To confirm that LacI-Rap1 tethering generates DNA breakage, we used an assay by combining TdT-mediated end-labeling and PCR (Fig 4C and S8 Fig). In this assay PCR amplification detects the addition of G-tracts at 3’-DNA ends near the LacO_16_ repeat. PCR amplified DNA fragments that correspond to DSB induction at the LacO_16_ repeat in both directions after LacI-Rap1 expression whereas no discrete band was observed after LacI expression, indicating that LacI-Rap1 expression results in DSB induction near the LacO_16_ repeat.

**Fig 5:**
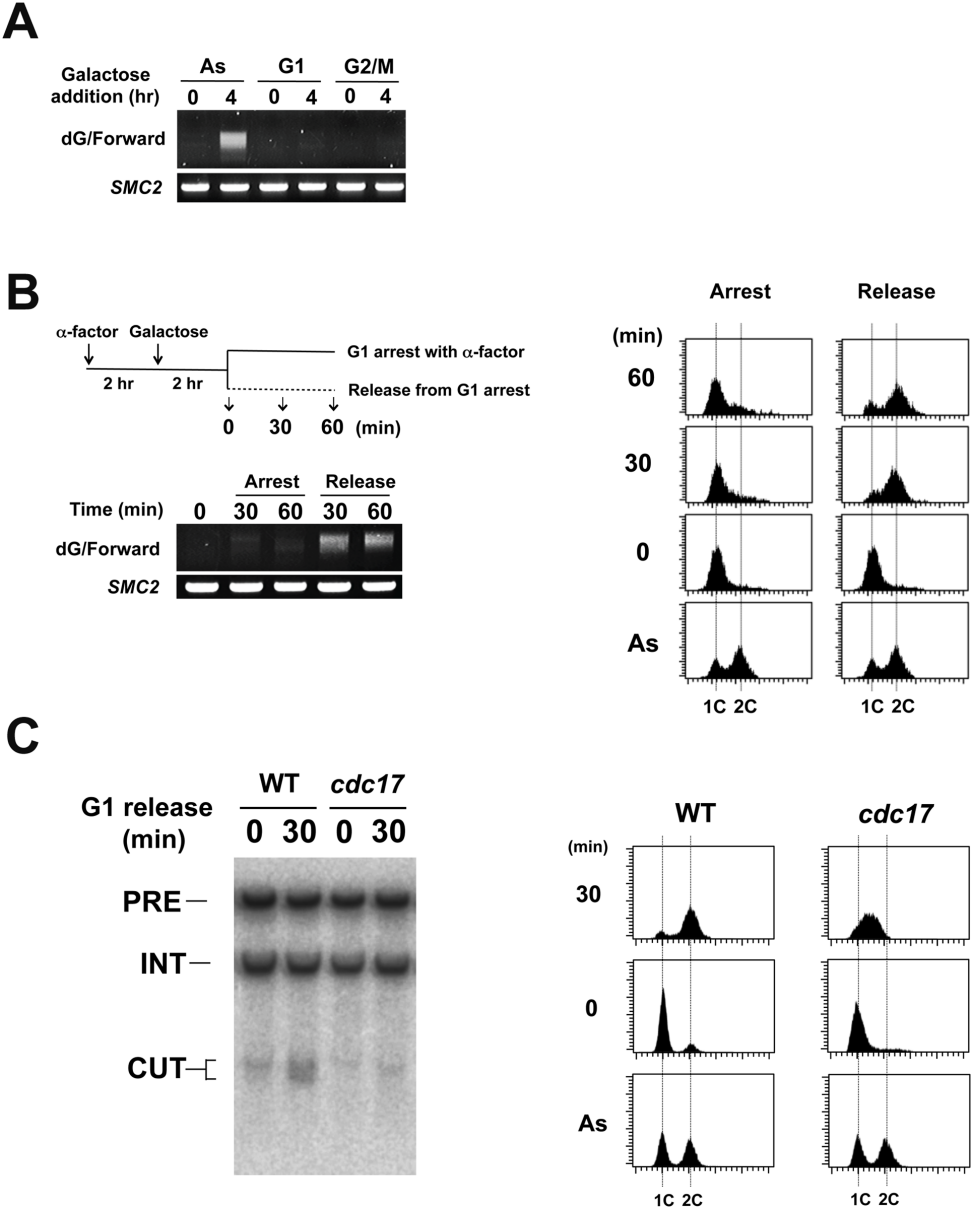
Effect of cell-cycle progression on Rap1-mediated DSB induction. (A) Effect of G1 or G2/M arrest on DNA breakage induction. LacO_16_-URA3 cells carrying pGAL-LacI-RAP1 were grown in 2% sucrose and 0.5% glucose and treated with α-factor or nocodazole for 2 hr. After arrest, galactose (final concentration 2%) was added to induce LacI-Rap1 expression. After 4 hr induction, cells were collected for genomic DNA preparation. DNA was analyzed by the TdT assay as in Fig 4C using the forward primer. (B) DNA breakage induction during S phase. LacO_16_-URA3 cells carrying pGAL-LacI-RAP1 were grown in 2% sucrose and 0.5% glucose and treated with α-factor. After arrest, galactose (final concentration 2%) was added to induce LacI-Rap1 expression. After 2 hr incubation of galactose, half of the culture was released from α-factor while another half was kept arrested at G1 with α-factor. G1 to S phase progression was monitored by flow cytometric analysis. Cells were collected at the indicated time point after release from α-factor. DNA was analyzed by the TdT assay as in Fig 4C. (C) Effect of *cdc17-1* mutation on DNA breakage induction. LacO_16_-URA3 *CDC17* or *cdc17-1* cells carrying pGAL-LacI-RAP1 were grown in 2% sucrose and 0.5% glucose and treated with α-factor at 25°C. After arrest, galactose (final concentration 2%) was added to induce LacI-Rap1 expression and the culture was shifted to 32°C. After 3 hr incubation of galactose, half of the culture was released from α-factor at 32°C while another half was kept arrested at G1 with α-factor. Cells were collected 30 min after release from α-factor. DNA was analyzed by Southern blotting as in Fig 4B. G1 to S phase progression was monitored by flow cytometric analysis.

We next addressed in which cell cycle stage LacI-Rap1 expression induces DNA breaks by the TdT-based PCR assay. Cells were grown in sucrose and treated with α-factor or nocodazole to synchronize in G1 or G2/M phase, respectively (Fig 5A). Synchronized cells were then incubated with galactose to induce LacI-Rap1 expression. No DSB induction was detected in G1 or G2/M-arrested cells. We next examined whether DNA breakage occurs in S phase (Fig 5B). Cells arrested with α-factor in G1 were incubated with galactose to express LacI-Rap1 fusion. Cells were then released from α-factor arrest or remained arrested. DNA flow cytometry analysis confirmed that cells underwent S phase after α-factor release (Fig 5B). DSB induction was detected in S phase after α-factor release but not in G1-arrested cells. Thus, LacI-Rap1 expression leads to DSB induction at the LacO_16_ repeat during S phase. To address whether DSB induction is coupled with DNA replication, we examined the effect of temperature-sensitive *cdc17-1* mutation on break induction during S phase (Fig 5C). *CDC17* encodes a catalytic subunit of DNA polymerase α. Wild-type or *cdc17-1* mutants carrying pGAL-LacI-RAP1 were arrested with α-factor in G1 and incubated with galactose at the restrictive temperature and then released from α-factor arrest. We then monitored DSB induction by Southern blotting analysis (Fig 5C). While wild-type cells underwent DNA replication, *cdc17-1* mutants arrested in early S phase (Fig 5C). The *cdc17-1* mutation suppressed DSB induction. These results show that replication fork progression in S phase is required for DSB induction.

**Fig 6:**
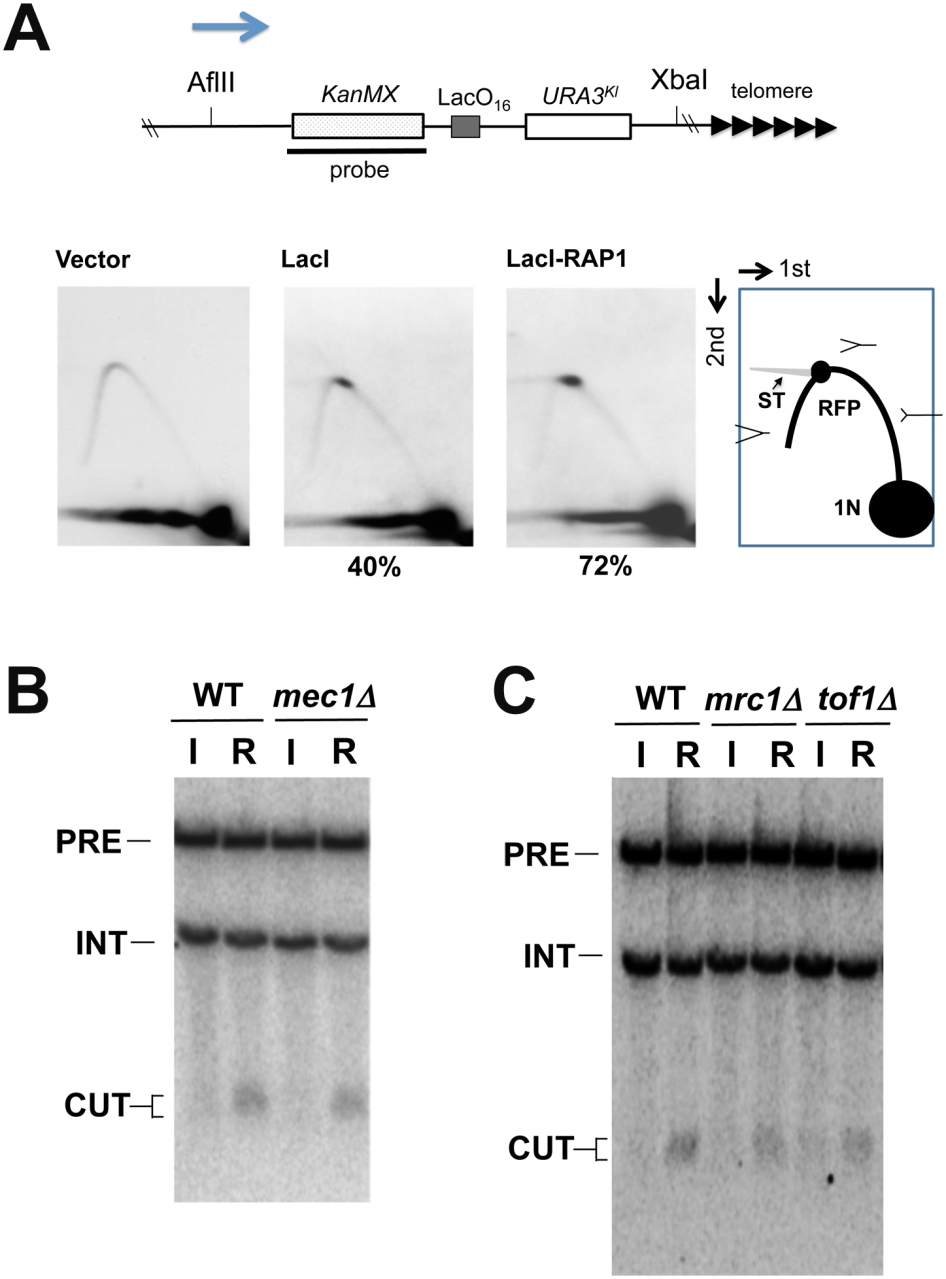
DNA replication fork pausing at the LacO_16_ repeat after LacI-Rap1 expression. (A) LacO_16_-URA3 cells carrying pGAL-LacI-RAP1, pGAL-LacI or the control vector were cultured as in Fig 4B. CsCl gradient purified DNA was digested with AflII and XbaI and analyzed by two-dimensional gel electrophoresis using the indicated probe. The probe detects a 6.3 kb AflII-XbaI fragment. The LacO_16_ repeat locates 3.0 kb from the AflII site and 2.9 kb from the XbaI site. RFP represents replication fork pausing. Note that some parts of RFP signal are smearing (ST). The number (%) below each panel denotes the ratio of the signal of RFP to that of total replication intermediates. The arrow indicates the direction of replication fork movement. There is a highly active replication origin 40 kb proximal to the LacO_16_ repeat insert site (the *ADH4* locus) on chromosome VII [82]. (B) Effect of *mec1Δ* mutation on DNA breakage induction. LacO_16_-URA3 *MEC1* or *mec1Δ* cells were transformed with pGAL-LacI (I) or pGAL-LacI-RAP1 (R). Transformants were cultured and DNA was analyzed as in Fig 4B. Cells contain an *sml1Δ* mutation. Hybridization detects two NcoI-fragments; one from chromosome I (5 kb, marked with INT) and the other from the chromosome VII region (11.6 kb, marked with PRE). DNA breakage at LacO_16_ generates a 1.3–1.7 kb fragment (marked with CUT) from the 11.6 kb fragment. (C) Effect of *mrc1Δ* or *tof1Δ* mutation on DNA breakage induction. LacO_16_-URA3, LacO_16_-URA3 *mrc1Δ* or LacO_16_-URA3 *tof1Δ* cells were transformed with pGAL-LacI or pGAL-LacI-RAP1. Transformants were cultured and DNA was analyzed as in (B).

We analyzed replication fork progression at the LacO_16_ locus after LacI-Rap1 or LacI expression by two-dimensional gel electrophoresis (Fig 6A and S9 Fig). Cells carrying pGAL-LacI-RAP1, pGAL-LacI, or the control vector were initially grown in sucrose and then incubated with galactose for 4 hr to induce LacI-Rap1 or LacI expression. There was no replication fork pausing in cells carrying the control vector. Experiments using two different sets of restriction enzymes confirmed that replication forks pause at the LacO_16_ repeat. Replication fork pausing was seen after both LacI expression and LacI-Rap1 expression but corresponding signals were only two-fold more intense in cells expressing LacI-Rap1 than in those expressing LacI alone. As described above, however, LacI-Rap1 expression resulted in DNA breakage much more robustly than LacI expression (Fig 4B and S7 Fig); LacI-Rap1 was estimated to promote DSB induction ~100-fold more strongly than LacI alone (Fig 3C). Thus, fork stalling per se did not fully explain the mechanism of DSB induction after LacI-Rap1 expression.

The ATR/Mec1 checkpoint pathway has been proposed to facilitate replication progression and prevent chromosome breakage during replication stress [42,53]. We tested the possibility that Rap1 impairs Mec1 function that prevents chromosome breakage (Fig 6B). If this were the case, LacI expression could generate DNA breaks as efficiently as LacI-Rap1 expression in *mec1Δ* mutants. However, DSB induction was still hardly detectable in *mec1Δ* mutants after LacI expression. Moreover, the introduction of *mec1Δ* mutation did not elevate DSB induction after LacI-Rap1 expression (Fig 6B). Mrc1 and Tof1 stabilize DNA replication forks and contribute to the activation of the Mec1 pathway during replication stress [54–57]. Previous studies have suggested that Mrc1 and Tof1 have a role in telomere stability in addition to fork progression [58–60]. We examined the effect of *mrc1Δ* or *tof1Δ* mutation on DNA break induction at LacO_16_ after LacI-Rap1 expression (Fig 6C). Neither *mrc1Δ* nor *tof1Δ* mutation increased the frequency of DSB induction. Thus, inactivation of Mec1 checkpoint function does not affect Rap1-induced DSB induction.

### Rap1-mediated DSB induction at artificially extended telomeres

**Fig 7:**
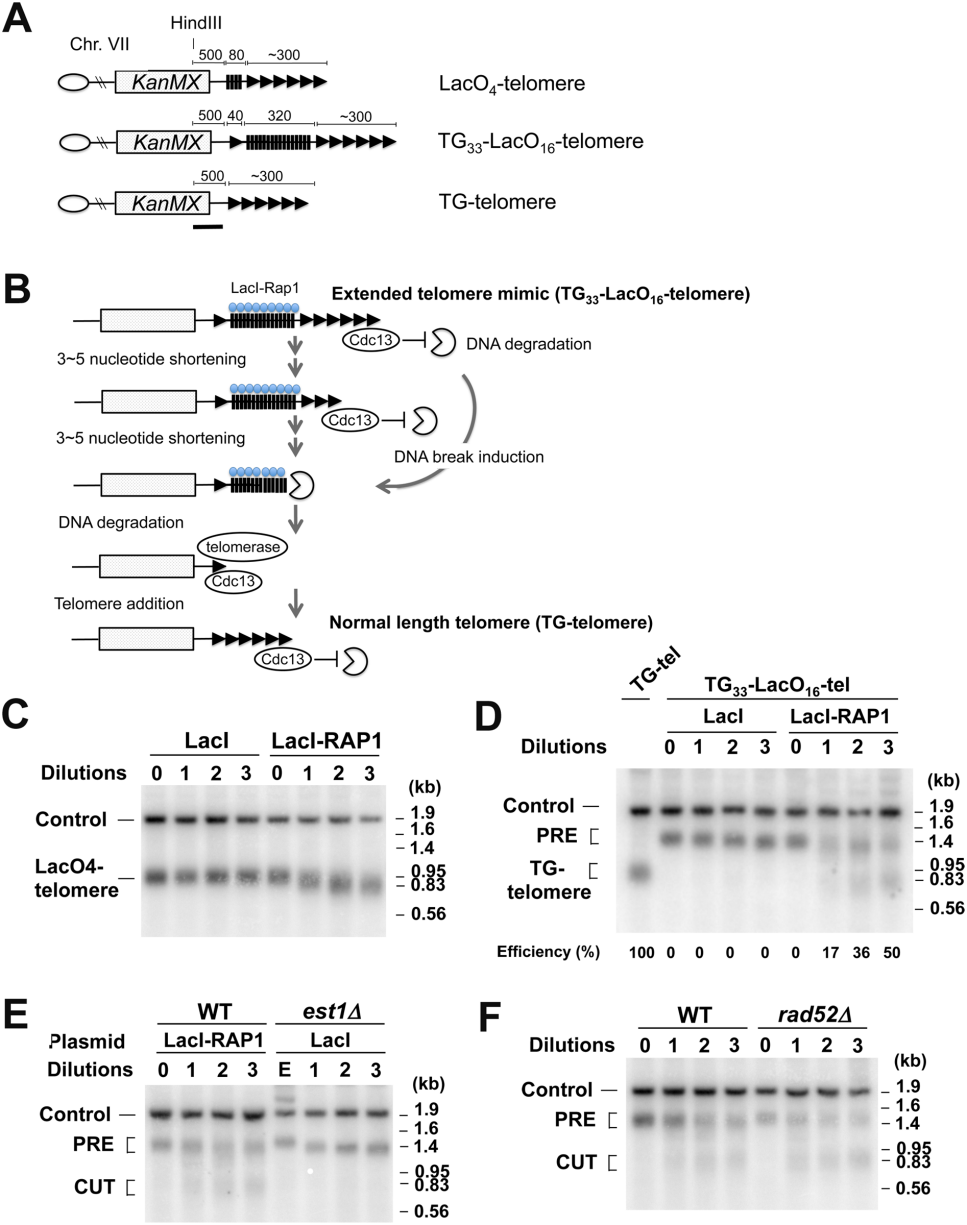
DSB induction at an artificially elongated telomere. (A) Schematic of modified VII-L telomeres. Telomeres marked the *KanMX* marker are generated near the *ADH4* locus. Triangles and black bars represent TG and lacO sequence, respectively. The VII-L subtelomere sequence is deleted. The black line indicates a hybridization probe. Each chromosome end contains a wild-type length telomere (~300 bp) before LacI-Rap1 expression. Drawing not to scale. (B) Schematic of experimental protocol to detect DNA breaks at an extended telomere. LacI-Rap1 expression converts TG_33_-LacO_16_-telomeres to a long telomere mimic. Telomeres become shortened 3–5 bp per generation because of the end-replication problem. Cdc13 binds single-stranded telomeric TG DNA and blocks DNA degradation. Telomere shortening or DSB induction needs to trim off ~300 bp telomeric TG sequence to initiate degradation of the LacO_16_ repeat. Once the LacO_16_ repeat is degraded, telomere addition occurs at the TG_33_ repeat, generating TG-telomere. (C) Effect of LacI-Rap1 expression on the length of LacO_4_-telomeres. Cells containing the LacO_4_-telomere were transformed with pGAL-LacI-RAP1 or pGAL-LacI. Transformed cells were grown in sucrose to full growth. The culture was then diluted 1000-fold in 2% galctose and 0.5% glucose to allow cells to undergo cell division for 24 hr and aliquots were collected for genomic DNA preparation. This cycle was repeated three times. DNA was digested with HindIII and analyzed by Southern blot using the probe shown in (A). The probe detects the LacO_4_-telomere and a 1.8 kb HindIII fragment (Control) from the *SMC2* locus on chromosome VI. (D) Effect of LacI-Rap1 expression on the length of TG_33_-LacO_16_-telomeres. Cells containing the TG_33_-LacO_16_-telomere (TG_33_-LacO_16_-tel cells) were transformed with pGAL-LacI-RAP1 or pGAL-LacI. Transformants were analyzed as in (C). As control, DNA from cells containing TG-telomere was examined together. The band labeled PRE indicates a fragment containing TG_33_-LacO_16_ telomere (~1.2 kb). After DSB induction within or near the LacO_16_ repeat, this band is converted to a new band (~0.8 kb), which is similar to the fragment containing wild-type length TG-telomeres. The probe also detects a 1.8 kb HindIII fragment (Control) from the *SMC2* locus on chromosome VI. (E) Effect of telomerase loss on the length of TG_33_-LacO_16_-telomeres. TG_33_-LacO_16_-tel cells were transformed with pGAL-LacI-RAP1 whereas TG_33_-LacO_16_-tel *est1Δ* cells carrying the *URA3*-marked *EST1* plasmid were transformed with pGAL-LacI. Transformants were streaked on plates containing 5-FOA and colonies of Ura^-^ cells were inoculated in 2% galactose and 0.5% glucose medium and grown to the late log phase (1st dilution). The culture was diluted 1000-fold to allow cells to undergo cell division for 24 hr. This cycle was repeated twice. DNA was analyzed as in (D). TG_33_-LacO_16_-tel cells in sucrose (0 dilution) or *est1Δ* mutants carrying the *URA3*-marked *EST1* plasmid (E) in sucrose were examined as control. (F) Effect of r*ad52Δ* mutation on the length of TG_33_-LacO_16_-telomeres after LacI-Rap1 expression. TG_33_-LacO_16_-tel wild-type or *rad52Δ* cells were transformed with pGAL-LacI-RAP1. Transformed cells were analyzed as in (D).

We addressed whether Rap1 mediates DSB induction at telomeric regions. If the above model were applied to telomeres, Rap1 binding could generate DNA breakage at extended telomeres and truncate them. In parallel, however, Rap1 inhibits telomerase recruitment at extended telomeres and negatively regulates their length. To distinguish between these two different types of telomere shortening, we developed a system that generates an artificially elongated telomere. In this system the LacO_16_ sequence is integrated between a 33 bp TG sequence (TG_33_) and the VII-L telomere (Fig 7A). Non-telomeric sequences can be counted as telomeric sequences if Rap1 is anchored [25]. Therefore, the TG_33_-LacO_16_-telomere becomes an extended telomere mimic after LacI-Rap1 expression (Fig 7B). Once converted to an extended telomere, telomeres become shorter gradually (3–5 nucleotides per generation) because of the end-replication problem. While telomeric TG sequence is retained, Cdc13-telomere capping blocks DNA degradation [2]. However, once telomere shortening reaches the LacO_16_ sequence after 60–90 generations, the DNA end loses Cdc13-dependent protection and exonuclease activities start degrading the LacO_16_ repeat. It is estimated that DNA degradation occurs at the rate of 4 kb/hour [61]. Previous studies showed that 33 bp TG repeat sequences act as a telomere seed *in vivo* [25,62]. We have shown that DNA ends with 22 bp of TG repeat nearby are efficiently converted to telomeres [63]. If DNA degradation reaches to TG_33_ repeat sequences, telomere extension occurs using TG_33_, generating TG-telomeres. However, DSB induction near or within LacO_16_ could skip gradual telomere shortening that results from the end-replication problem. Thus, DSB induction can be detected as swift conversion from the TG_33_-LacO_16_-telomere to TG-telomere. As mentioned above, there is no putative G4 forming sequence on the LacO repeat sequences. This system therefore enables us to examine the effect of Rap1 binding on DSB induction without increasing the length of TG repeat sequence that potentially generates G4 structures.

We first confirmed that targeted LacI-Rap1 to the LacO_4_ repeat behaves in a manner identical to Rap1 with respect to telomere length control. We used a strain with the LacO_4_ repeat adjacent to VII-L telomere (LacO_4_-telomere). Previous studies showed that the GAL4 binding site adjacent to telomeres behaves in a manner similar to a telomere sequence after expression of a Gal4-Rap1 fusion protein [25]. Cells containing the LacO_4_-telomere were transformed with pGAL-LacI-RAP1 or pGAL-LacI and cultured in sucrose to stationary phase. The cultures were diluted 1,000-fold in galactose and grown for 24 hr (eight generations) for successive serial dilutions. Cells were collected at each dilution point and the length of LacO_4_-telomere was monitored by Southern blot analysis (Fig 7C). When LacI-Rap1 was expressed, the length of the telomere repeat tract was gradually decreased as telomerase activity is down regulated at longer telomeres [25]. In contrast, the expression of LacI alone did not have any effect on telomere length. LacO_4_-telomeres became about 80 bp shorter after the serial dilution culture (Fig 7C). Thus, the lacO array sequence is counted as a part of the telomere after LacI-Rap1 expression, supporting the hypothesis that the TG_33_-LacO_16_-telomere with LacI-Rap1 coated on the lacO sequence behaves like an extended telomere.

We then examined the effect of LacI-Rap1 or LacI expression on the length of TG_33_-LacO_16_-telomeres. Cells containing TG_33_-LacO_16_-telomere carrying pGAL-LacI-RAP1 or pGAL-LacI were cultured as above and telomere length was monitored by Southern blot analysis (Fig 7D). As LacI expression had no impact on the length of LacO_4_-telomeres (Fig 7C), it did not affect the length of TG_33_-LacO_16_-telomere. In contrast, if LacI-Rap1 was expressed, two different types of telomere shortening were observed. First, TG_33_-LacO_16_ telomeres (PRE) became gradually shorter consistent with the above finding that LacI-Rap1-covered lacO sequence is counted as a telomere sequence. This observation indicates that telomerase activity is inhibited because TG_33_-LacO_16_ telomeres behave like an extended telomere in the presence of LacI-Rap1. Second, LacI-Rap1 expression generated shorter telomeres (TG-telomeres) corresponding to those extended from the TG_33_ repeat, consistent with the hypothesis that DSB induction occurs near or within the LacO_16_ repeat. As Rap1-mediated DSB induction occurs during S phase, continuous culturing increased the population size of cells containing TG-telomeres. 50% of the cells converted from the TG_33_-LacO_16_ telomere to the TG-telomere after three successive dilutions (24 generations); it was estimated that 2% of cells received a DNA break within or adjacent to the LacO_16_ repeat per generation.

To confirm that the rapid generation of TG-telomeres does not result from the inhibition of telomerase activity, we compared telomere shortening after LacI-Rap1 expression and telomerase depletion (Fig 7E). We introduced a deletion mutation of *EST1*, which encodes a regulatory protein for telomerase, in cells containing the TG_33_-LacO_16_ telomere. Growth of the *est1Δ* strains was maintained by wild-type *EST1* on a *URA3*-marked plasmid. Wild-type cells and *est1Δ* mutant cells were transformed with pGAL-LacI-RAP1 and pGAL-LacI, respectively. To select for loss of the complementing *EST1* plasmid, cells were grown on sucrose plates containing 5-FOA. The resulting single colonies were inoculated into galactose medium (1st dilution). After 24 hr culture, it was diluted 1000-fold for successive serial dilutions. Southern blotting analysis confirmed that the inhibition of telomerase activity caused slow telomere shortening (~30 bp during each dilution) but did not promote TG-telomere formation (Fig 7E). As discussed above, the generation of TG-telomeres was dependent on telomerase activity (S10 Fig). To exclude the possibility that the emergence of TG-telomeres results from homologous recombination among telomeric sequences, we tested the effect of *rad52Δ* mutation on shortening of the TG_33_-LacO_16_-telomere after LacI-Rap1 expression (Fig 7F). Cells were first grown in sucrose and cultured continuously in galactose to express LacI-Rap1 after serial dilutions. The *rad52Δ* mutation did not affect shortening of TG_33_-LacO_16_ telomeres after LacI-Rap1 expression. Thus, it is unlikely that Rap1 binding stimulates homologous recombination between telomeric TG repeat sequences. Collectively, our results support the model in which Rap1 binding is involved in telomere shortening by introducing DNA breaks.

## Discussion

Rap1 binds to double-stranded telomeric TG-repeat sequences and recruits Rif1 and Rif2 proteins via its C-terminal domain [21–24]. Previous studies have uncovered a negative-feedback mechanism that counts telomere-binding Rap1 protein to control telomere length [25,26]. In this feedback loop, Rap1 collaborates with Rif1 and Rif2 and inhibits the localization of the protein kinase Tel1 to adjacent DNA ends, thereby attenuating the recruitment of telomerase to long telomeres [30–32,34–36]. In this report we have provided evidence suggesting that an alternative Rap1-dependent mechanism operates to trim elongated telomeres. In this telomere shortening mechanism, Rap1 binding coupled with DNA replication promotes DSB induction independently of Rif1 or Rif2. These observations support a model in which Rap1 negatively regulates telomere length through two distinctive mechanisms.

DNA breaks adjacent to telomeric TG repeats can be readily converted to telomeres by telomerase [38,40]. It is therefore complicated to detect DNA breaks at telomeres. We have developed several experimental systems to monitor DSB induction and shown that binding of multiple Rap1 proteins induces DNA breaks at both telomeric and non-telomeric regions. Rap1-mediated DSB induction appears to operate in a dosage-dependent manner. First, longer TG repeats trigger chromosome truncation more efficiently than shorter TG repeats. Second, LacI-Rap1 expression promotes nearby recombination at longer lacO arrays more frequently than at shorter ones. As discussed above, the LacO_16_ repeat in the presence of LacI-Rap1 could correspond to wild-type length telomere in terms of Rap1 binding. To detect DSB induction at telomeres, we used TG_33_-LacO_16_-telomeres, which correspond to ~600 bp long telomeres in the presence of LacI-Rap1. Two percent of TG_33_-LacO_16_-telomeres were estimated to receive DNA breaks near or within the LacO_16_ repeat every cell division in the presence of LacI-Rap1. Although the frequency is low (2% per generation), approximately 50% of cells would receive DNA breaks after 32 generations. DNA ends adjacent to TG repeats, once generated, are protected from DNA degradation by Cdc13-mediated telomere capping [2,35]. Thus, Rap1-mediated DSB induction at telomeres seems to be a physiological phenomenon that keeps telomeres in normal-length ranges, although it remains possible that telomere-specific activity suppresses DSB induction at native telomeres. Rap1 collaborates with Rif1 and Rif2 to inhibit telomerase recruitment [64]. In contrast, Rif1 and Rif2 are dispensable for Rap1-mediated DSB induction. Telomerase inhibition steadily shortens telomeres 3–5 bp per cell division because of the end-replication problem [6,9]. In contrast, DSB induction can delete longer telomere sequence although it may not constantly occur. The extent of telomerase-dependent telomere extension varies at each telomere and is independent of telomere length [65]. Cells take full advantage of different telomere shortening modes to cope with the heterogeneous nature of telomeres.

Replication forks in yeast and other organisms move more slowly through telomeric DNA than non-telomeric regions [43,66–68]. This replication problem is thought to result from the G-rich nature of telomeric DNA, which allows it to form G4 DNA. G4 DNA interferes with DNA replication and generates DNA breaks [37]. In addition to G4 structures, we have shown that binding of multiple Rap1 proteins generates a barrier that stimulates DSB induction during DNA replication. Persistent fork stalling could lead to fork collapse and subsequent DSB induction [42]. Consistent with this model, tight binding of LacI was critical for LacI-Rap1-mediated DSB induction. However, fork stalling by itself does not appear to result in DSB induction at Rap1 bound regions. We found that LacI-Rap1 expression induced DSB formation about 100-fold more efficiently than LacI expression alone whereas the rate of fork stalling after LacI-Rap1 expression was only two-fold higher compared with LacI expression alone. Moreover, larger complex formation with Rif1, Rif2, Sir3 and Sir4 did not increase the frequency of DSB induction. It seems less likely that DSB induction results from DNA bending because the N-terminus is dispensable [19]. Several lines of evidence have shown that paused forks by themselves are relatively stable [69]. Indeed, 40% of the replicating intermediates contained pausing forks at the LacO_16_ repeat after LacI expression but 0.02% of cells were expected to receive DNA breaks at the LacO_16_ repeat per generation. The ATR/Mec1 checkpoint pathway has been proposed to prevent chromosome breakage during replication stress [42,53]. However, inactivation of the Mec1 checkpoint pathway did not affect DSB induction after LacI or LacI-Rap1 expression. Thus, Rap1 appears to impair other mechanisms than Mec1 checkpoint function at DNA replication forks. We found that the overall structure of the central Rap1 region plays an important role in DSB induction. It has been shown that the central region of Rap1 inhibits NHEJ at telomeres [70]. This Rap1 region, once arrayed, might disrupt replication complexes as well as repair machinery along the DNA tract. We note that not all telomere-binding proteins have a negative impact on DNA replication. Taz1 in fission yeast and TRF1 in human have been shown to promote DNA replication at telomeres [67,68]. Interestingly, Taz1 and TRF1 possess a Myb domain as does the Rap1 central region. The central region of Rap1 also stimulates DSB induction for meiotic recombination [46]; however, meiotic DSB induction occurs through a different mechanism. Transcription factors including Rap1 generate nucleosome free regions where Spo11 binds to chromatin and catalyzes DSB [71].

Over-elongated telomeres can be shortened to normal length through a mechanism termed telomeric rapid deletion (TRD) [10]. TRD appears to result from an intra-chromosomal recombination event between telomeric repeats because TRD depends on the major recombination protein, Rad52 [10]. In addition, *hpr1* mutation, which increases recombination between direct repeats, elevates the rate of TRD [10]. Similar telomere shortening has been observed in other systems including human cell lines and most likely involves homologous recombination-mediated removal of telomere loops [11–13]. While TRD largely depends on Rad52 function, some fraction of TRD was found to occur in a Rad52-independent manner [10]. Since DSBs near telomeric TG sequences are healed by telomere addition, DSB generation at over-elongated telomeres would lead to Rad52-independent TRD. G4 structure formation could promote DSB induction at telomeres [37], thereby trimming telomeres independently of Rad52 function. However, our results suggest that Rap1-mediated DSB induction contribute to Rad52-independent TRD as well. Indeed, TG_33_-LacO_16_-telomeres became shorter independently of Rad52 function or the end-replication problem after LacI-Rap1 expression.

In summary, we have provided evidence indicating that binding of multiple Rap1 proteins promotes DNA break induction during DNA replication. Since Rap1 binds extensively at telomeric DNA regions [72], it seems likely that Rap1 binding promotes DSB induction at telomeres. Rap1 binds to telomeres and controls their function in other eukaryotes [73–75]. Given that telomeres consist of repetitive sequences and sequence-specific binding proteins, a similar system may function in other organisms as well.

## Materials and Methods

### Strains

The strain carrying the TG_0_-URA3, TG_81_-URA3, TG_250_-URA3 or LacO_16_-URA3 cassette was generated by the pNO-URA3, pT81-URA3, pT250-URA3 or pO16-URA3 plasmid after digestion with NotI and SalI, respectively. The strain carrying the LacO_0_-ura3-ΔC, LacO_4_-ura3-ΔC, LacO_8_-ura3-ΔC or LacO_16_-ura3-ΔC or TetO_8_-ura3-ΔC cassette was generated by the pUN-O0, pUN-O4, pUN-O8, pUN-O16 or pUN-tetO8 plasmid after digestion with EcoRI and SalI, respectively. The strain containing the LacO_4_-telomere, TG_33_-LacO_16_-telomere, or TG-telomere was generated by the pO4-TG-HO, pTG33-O16-TG-HO or pTG_81_-HO plasmid after digestion with EcoRI and SalI, respectively. The ura3-ΔN-Hph cassette was introduced into the *YER186* locus by a PCR-based method [76]. The copper-inducible RAP1 degron (*rap1-(Δ)*) construct has been described [44]. The N-terminal deletion of RAP1 (*rap1-ΔN*) has been described [46]. TG-telomere cells were generated from TG_81_-HO cells after HO expression [35](See S1 Fig). The *mec1Δ*, *rad52Δ* or *sml1Δ* mutation has been described [77]. The *mec1-81* or *tel1Δ* mutation has been published [29]. The *est1Δ* mutation has been described [78]. The C-terminal *rap1* truncation (*rap1-ΔC*), *rif1Δ* and *rif2Δ* mutation have been described [35]. The *rap1-ΔC* allele encodes the same truncated Rap1 proteins as the *rap1-17* mutation does [26,45]. Disruption of *SIR3* was performed as described [79]. The copper inducible protein degradation system was described [80]. The *SIR4* disruption plasmid was obtained from Masayasu Nomura. The strains used in this study are listed in S1 Table.

### DNA preparation and hybridization probes for Southern blot analysis

The probe that detects the *KanMX* gene was obtained by NotI-digestion of the pFA6-kanMX4 plasmid [79]. The probe that detects DSB induction was a PCR fusion product of the *KanMX* coding sequence and the *LTE1* locus, which were amplified by the primer pair KSX050/89 and KS3004/3005, respectively. The probe that detects endogenous telomeres or telomere addition at the *ADH4* locus has been described [35,78]. DNA probes were DIG-dUTP—or ^32^P-labeled by using the DIG prime (Roche) or the Random Primer DNA Labeling Kit (Clontech), respectively. Genomic DNA was purified using a MasterPure yeast DNA purification kit (Epicentre).

### TG-mediated *URA3* marker loss

Cells from a single colony were fully grown in uracil dropout medium. The culture was then diluted 1000-fold and grown in rich medium for 24 hr. Aliquots of the cultures were diluted and plated on plates containing 5-FOA or non-selectable rich medium. Rates per generation were calculated using the FALCOR program based on the Luria-Delbruck fluctuation analysis [81]. We confirmed that essentially all 5-FOA resistant cells derived from TG_81_-URA3 or TG_250_-URA3 are Ura^-^ cells. The promoter of *URA3* ^*Kl*^ was replaced with the strong *ADH1* promoter (see S1 Text). More than 500 colonies of each TG_81_-URA3, TG_250_-URA3, TG_250_-URA3 *rad52Δ* or TG_250_-URA3 *rap1-(Δ)* cells on 5-FOA plates were tested by replica-plating to uracil drop-out plates. None of them grew on uracil-drop out plates.

### *URA3* recombination assay

Cells were transformed with *TRP1*-marked plasmids and grown in tryptophan-dropout medium containing 2% sucrose overnight. The culture was then diluted and grown in tryptophan-dropout medium containing 2% galactose or 2% sucrose and 0.5% glucose. Aliquots of the cultures were diluted and plated on uracil-dropout or rich medium to estimate the *URA3* recombination frequency after expression of LacI or LacI-fusion protein. Rates per generation were calculated using the FALCOR program [81].

### DNA end detection by dC-tailing

Purified genomic DNA (100 ng) was incubated with one unit of terminal deoxynucleotidyl-transferase (TdT; New England BioLabs) in a supplied reaction buffer supplemented with 0.2 mM dCTP at 37°C for 60 min, followed by PCR using a poly(dG)-oligonucleotide with either the TdT forward or reverse primer. The PCR condition was 33 cycles of denaturation at 94°C for 30 s, annealing at 62°C for 30 s, and elongation at 72°C for 60 s. Sequences of PCR primers are described in S2 Table.

### Other methods

Immunoblotting or DNA flow cytometric analysis was performed as described [35,78]. Rap1 was detected with affinity-purified antibody (gift from V. Zakian, Princeton University) or antibody against the C-terminus (yC-19, Santa Cruz biotechnology). LacI fusions were detected with anti-LacI antibodies (clone 9A5, Millipore). Two-dimensional gel electrophoresis was performed in Tris-borate-EDTA as previously described [43]. Details of plasmid construction are described in S1 Text.

## Supporting Information

S1 Fig Telomere addition at TG repeats after URA3 marker loss. Cells containing the TG_81_ or TG_250_ cassette were treated as in Fig 1 and cells (ten of each) that grew on 5-FOA plates were subjected to Southern blotting analysis as in Fig 7. TG-telomere (T) cells were generated from TG_81_-HO cells after HO expression [35]. HO endonuclease induces a DSB break at the HO cassette.
(TIFF)

S2 Fig Effect of TG_250_ insertion on replication fork pausing. Cells containing the TG_0_ or TG_250_ cassette were cultured as in Fig 1E. CsCl gradient purified DNA was digested with SacI and PvuII and analyzed by two-dimensional gel electrophoresis using the indicated probe. The probe detects a 5.5 kb SacI-PvuII fragment. The TG_250_ repeat is located 3.2 kb from the SacI site and 1.9 kb from the PvuII site. RFP represents replication fork pausing. The arrow indicates the direction of replication fork movement.
(TIFF)

S3 Fig Complementation of the *rap1* deletion by LacI-Rap1 expression. *rap1Δ* cells carrying YCp-RAP1 (URA3) were transformed with YEp-RAP1, pGAL-LacI-RAP1 or the control vector (YCplac22). Transformants were streaked on plates containing 2% galactose, 0.5% glucose with (+) or without 5-FOA (-).
(TIFF)

S4 Fig Expression levels of LacI fusion proteins. (A) Expression level of LacI-Rap1 and Rap1. Cells carrying pGAL-LacI-RAP1 or the control vector were grown in 2% galactose and 0.5% glucose and analyzed by immunoblotting with anti-Rap1 antibodies. Tubulin was detected as a loading control. (B) Expression level of LacI-Rap1 and LacI. Cells carrying pGAL-LacI-RAP1 or pGAL-LacI were grown in 2% galactose and 0.5% glucose and analyzed by immunoblotting with anti-LacI antibodies. Tubulin was detected as a loading control. The asterisk indicates cross-reactive proteins. (C) Expression level of LacI-Rap1 and LacI-Rap1 (224–663). Cells carrying pGAL-LacI-RAP1 or pGAL-LacI-RAP1 (224–663) were analyzed as in (B). (D) Expression level of LacI-Rap1 and LacI-GAL4. Cells carrying pGAL-LacI-RAP1 or pGAL-LacI-GAL4 were analyzed as in (B). (E) Expression level of LacI**-Rap1. Cells carrying pGAL-LacI-RAP1 or pGAL-LacI**-RAP1 were analyzed as in (B).
(TIFF)

S5 Fig Effect of TetR-Rap1 on homologous recombination near the TetO_8_ cassette. Cells (*KanMX-ura3-ΔN-TetO*_*8*_, *HphMX-ura3-ΔC*) carrying YCpT-TetR, YCp-TetR-RAP1 or the control vector were cultured in medium selectable for the *TRP1* marker and examined as in Fig 3B. Number in parentheses indicates rate relative to cells containing the TetO_8_ cassette and carrying the control vector. The *ADH4* locus on chromosome VII was replaced with the *KanMX*-marked cassette containing the *ura3-ΔC* ^*Kl*^ truncated gene and eight copies of the tetO sequence.
(TIFF)

S6 Fig Requirement of Myb or TA domain for Rap1-mediated DNA break induction. (A) Deletion of Myb or TA domain in the LacI-RAP1 construct. Rap1 contains a BRCT domain, two Myb domains, and a transcription activation (TA) domain and a C-terminal RCT domain. Fusion proteins contain the DNA-binding domain of LacI (black square) with a nuclear localization signal. (B) Effect of Myb domain or TA domain deletion on homologous recombination near the LacO_16_ cassette. Cells carrying pGAL-LacI, pGAL-LacI-RAP1, or its derivatives were cultured and examined as in Fig 3B. Number in parentheses indicates rate relative to cells containing the LacO_16_ cassette and carrying the control vector. (C) Expression level of fusion proteins. Cells carrying pGAL-LacI-RAP1 or various deletion constructs were grown in 2% galactose and 0.5% glucose and analyzed by immunoblotting with anti-LacI antibodies. Tubulin was detected as a loading control.
(TIFF)

S7 Fig Quantification of Rap1-mediated DNA break induction by Southern blot. Cells containing the LacO_16_-URA3 cassette were transformed with pGAL-LacI-RAP1 (R) or pGAL-LacI (I). Transformants were initially grown in 2% sucrose and then incubated with 2% galactose and 0.5% glucose for 4 hr. Genomic DNA from cells expressing LacI or LacI-Rap1 was digested with NcoI (Lane 1–2). The LacO_16_ repeat is cloned between the BamHI and EcoRI sites (see also Fig 4A). Genomic DNA from the control cells carrying the control vector was digested with either NcoI and BamHI or NcoI and EcoRI, and serially diluted (Lane 3–10). Digested DNA was analyzed by Southern blot as in Fig 4B. It was estimated that 3% of cells received DNA breaks at the LacO_16_ locus after LacI-Rap1 expression by using a Typhoon imaging system.
(TIFF)

S8 Fig Quantification of Rap1-mediated DNA break induction by the TdT-PCR assay. LacO_16_-URA3 cells carrying pGAL-LacI-RAP1 were treated as in Fig 4C (Lane 1). HO cells carrying pGAL-HO were precultured in sucrose and then incubated with 2% glucose (D) to repress HO expression (Lane 2) or 2% galactose (G) to induce HO expression (Lane 3–7) for 4 hr. Genomic DNA was extracted and examined by the TdT-PCR assay as in Fig 4C. Samples from HO expressing cells were serially diluted before PCR (Lane 3–7). 80% of HO cells were found to induce a DNA break at the HO recognition site after HO induction [78]. It was estimated that 3% of LacO_16_-URA3 cells received DNA breaks with the LacO_16_ repeat sequence after LacI-Rap1 expression.
(TIFF)

S9 Fig Effect of LacI-Rap1 expression on replication fork pausing at the LacO_16_ repeat. CsCl gradient purified DNA was digested with SacI and PvuII and analyzed by two-dimensional gel electrophoresis using the indicated probe. The probe detects a 5.5 kb SacI-PvuII fragment. The LacO_16_ repeat is located 3.2 kb from the SacI site and 1.9 kb from the PvuII site. RFP represents replication fork pausing. Note that some parts of RFP signal are smearing (ST). The number (%) below each panel denotes the ratio of the signal of RFP to that of total replication intermediates. The arrow indicates the direction of replication fork movement.
(TIFF)

S10 Fig Effect of telomerase loss on generation of TG-telomeres from TG_33_-LacO_16_-telomeres. TG_33_-LacO_16_-tel cells and TG_33_-LacO_16_-tel *est1Δ* cells carrying the *URA3*-marked *EST1* plasmid were transformed with pGAL-LacI-RAP1. Transformants were treated and examined as in Fig 7E.
(TIFF)

S1 Table Strains used in this study. All the strains are isogenic and the detailed construction is described in Materials and Methods.
(DOCX)

S2 Table Oligonucleotides used in this study. (DOCX)

S1 Text Plasmids used in this study. Details of plasmid construction and plasmid information are described.
(DOCX)
