# Supplementary figures and images for: Binding of Multiple Rap1 Proteins Stimulates Chromosome Breakage Induction during DNA Replication
Source: PLoS Genet. 2015 Aug 11;11(8):e1005283. doi: 10.1371/journal.pgen.1005283 (PMC4532487; doi:10.1371/journal.pgen.1005283)

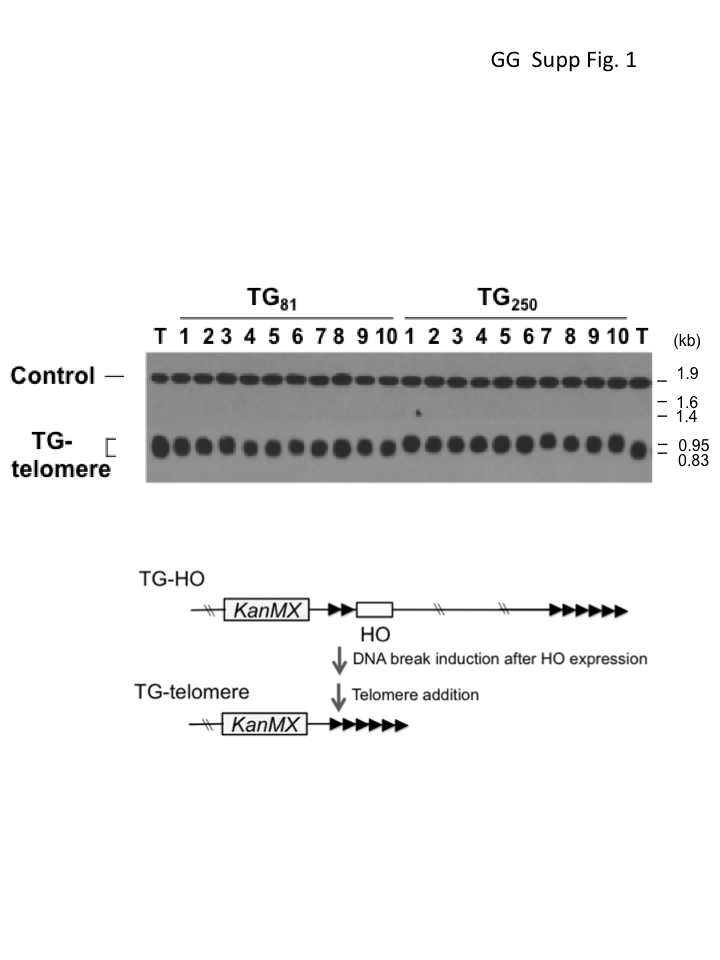

Supplement: S1 Fig — Cells containing the TG81 or TG250 cassette were treated as in Fig 1 and cells (ten of each) that grew on 5-FOA plates were subjected to Southern blotting analysis as in Fig 7. TG-telomere (T) cells were generated from TG81-HO cells after HO expression [35]. HO endonuclease induces a DSB break at the HO cassette. (TIFF) [file pgen.1005283.s001.tiff]

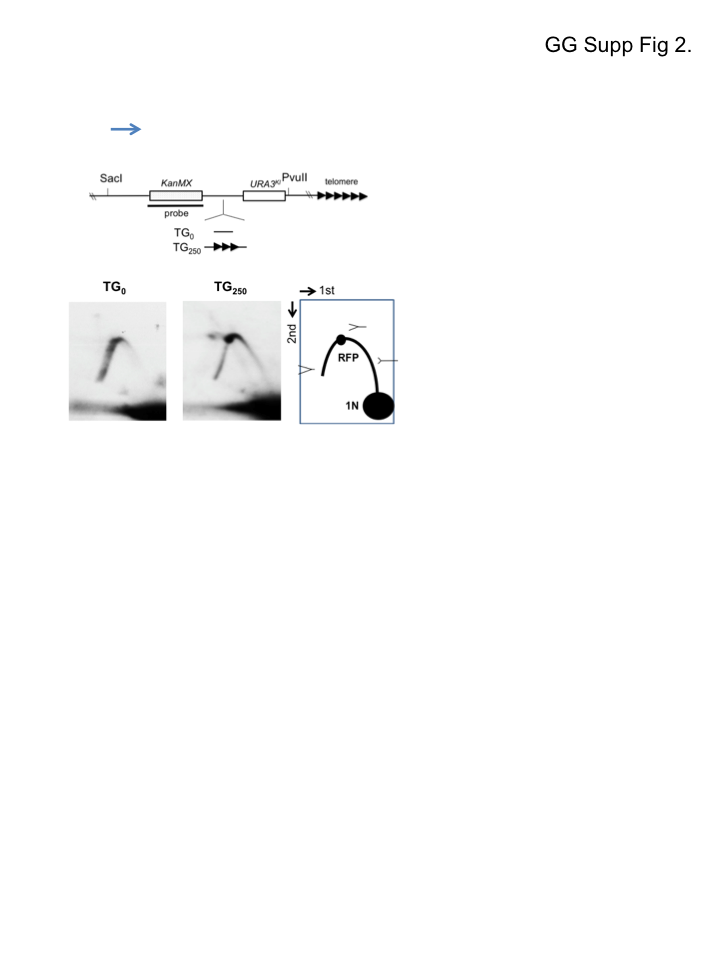

Supplement: S2 Fig — Cells containing the TG0 or TG250 cassette were cultured as in Fig 1E. CsCl gradient purified DNA was digested with SacI and PvuII and analyzed by two-dimensional gel electrophoresis using the indicated probe. The probe detects a 5.5 kb SacI-PvuII fragment. The TG250 repeat is located 3.2 kb from the SacI site and 1.9 kb from the PvuII site. RFP represents replication fork pausing. The arrow indicates the direction of replication fork movement. (TIFF) [file pgen.1005283.s002.tiff]

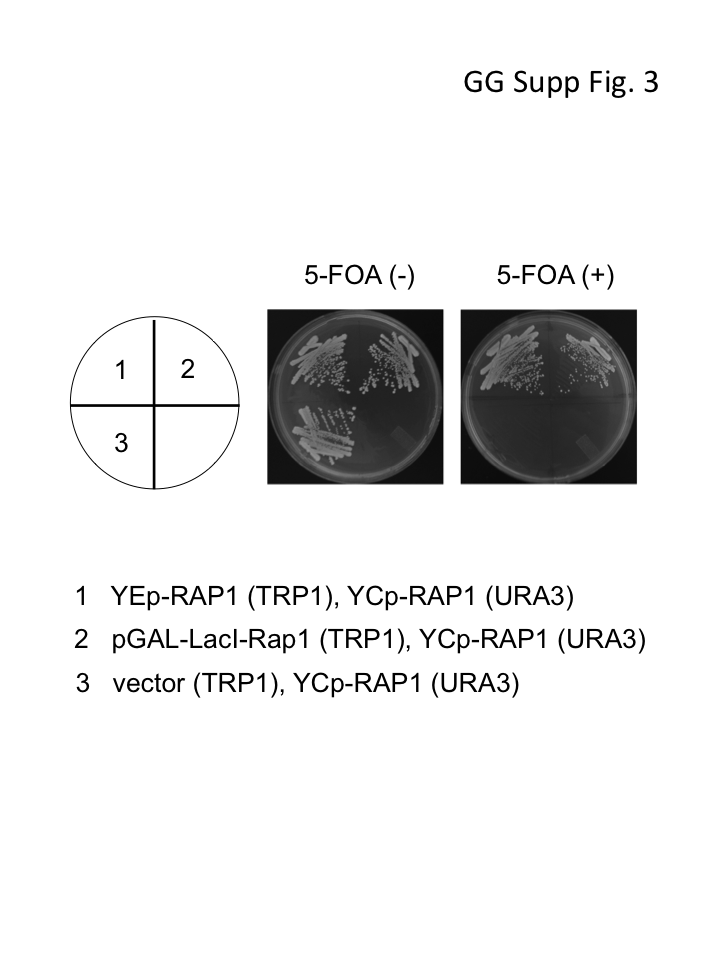

Supplement: S3 Fig — rap1Δ cells carrying YCp-RAP1 (URA3) were transformed with YEp-RAP1, pGAL-LacI-RAP1 or the control vector (YCplac22). Transformants were streaked on plates containing 2% galactose, 0.5% glucose with (+) or without 5-FOA (-). (TIFF) [file pgen.1005283.s003.tiff]

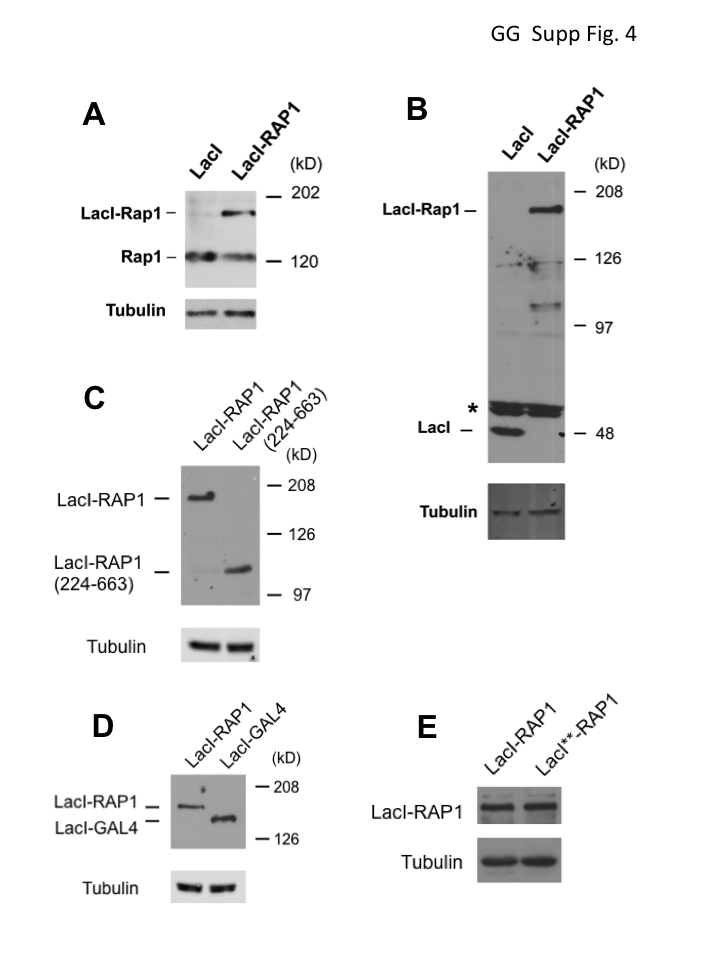

Supplement: S4 Fig — (A) Expression level of LacI-Rap1 and Rap1. Cells carrying pGAL-LacI-RAP1 or the control vector were grown in 2% galactose and 0.5% glucose and analyzed by immunoblotting with anti-Rap1 antibodies. Tubulin was detected as a loading control. (B) Expression level of LacI-Rap1 and LacI. Cells carrying pGAL-LacI-RAP1 or pGAL-LacI were grown in 2% galactose and 0.5% glucose and analyzed by immunoblotting with anti-LacI antibodies. Tubulin was detected as a loading control. The asterisk indicates cross-reactive proteins. (C) Expression level of LacI-Rap1 and LacI-Rap1 (224–663). Cells carrying pGAL-LacI-RAP1 or pGAL-LacI-RAP1 (224–663) were analyzed as in (B). (D) Expression level of LacI-Rap1 and LacI-GAL4. Cells carrying pGAL-LacI-RAP1 or pGAL-LacI-GAL4 were analyzed as in (B). (E) Expression level of LacI**-Rap1. Cells carrying pGAL-LacI-RAP1 or pGAL-LacI**-RAP1 were analyzed as in (B). (TIFF) [file pgen.1005283.s004.tiff]

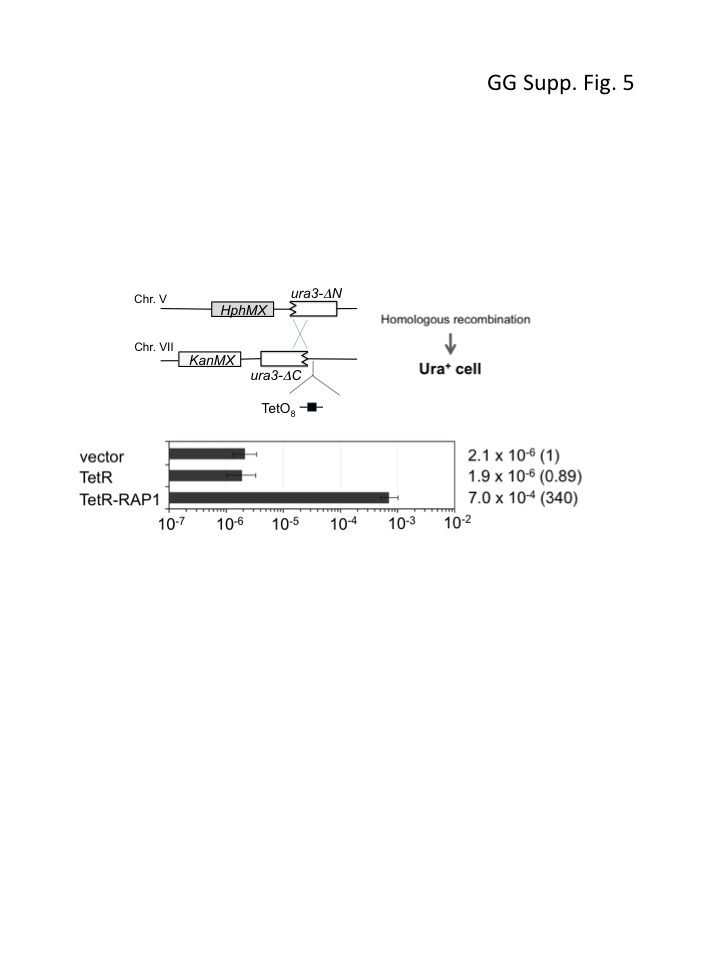

Supplement: S5 Fig — Cells (KanMX-ura3-ΔN-TetO 8, HphMX-ura3-ΔC) carrying YCpT-TetR, YCp-TetR-RAP1 or the control vector were cultured in medium selectable for the TRP1 marker and examined as in Fig 3B. Number in parentheses indicates rate relative to cells containing the TetO8 cassette and carrying the control vector. The ADH4 locus on chromosome VII was replaced with the KanMX-marked cassette containing the ura3-ΔC Kl truncated gene and eight copies of the tetO sequence. (TIFF) [file pgen.1005283.s005.tiff]

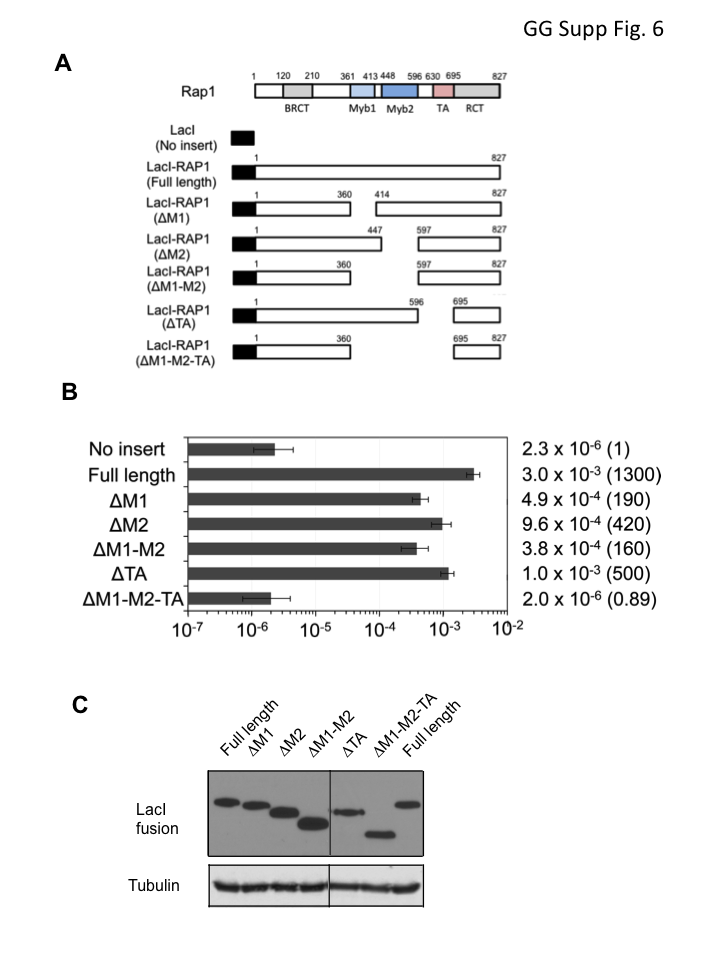

Supplement: S6 Fig — (A) Deletion of Myb or TA domain in the LacI-RAP1 construct. Rap1 contains a BRCT domain, two Myb domains, and a transcription activation (TA) domain and a C-terminal RCT domain. Fusion proteins contain the DNA-binding domain of LacI (black square) with a nuclear localization signal. (B) Effect of Myb domain or TA domain deletion on homologous recombination near the LacO16 cassette. Cells carrying pGAL-LacI, pGAL-LacI-RAP1, or its derivatives were cultured and examined as in Fig 3B. Number in parentheses indicates rate relative to cells containing the LacO16 cassette and carrying the control vector. (C) Expression level of fusion proteins. Cells carrying pGAL-LacI-RAP1 or various deletion constructs were grown in 2% galactose and 0.5% glucose and analyzed by immunoblotting with anti-LacI antibodies. Tubulin was detected as a loading control. (TIFF) [file pgen.1005283.s006.tiff]

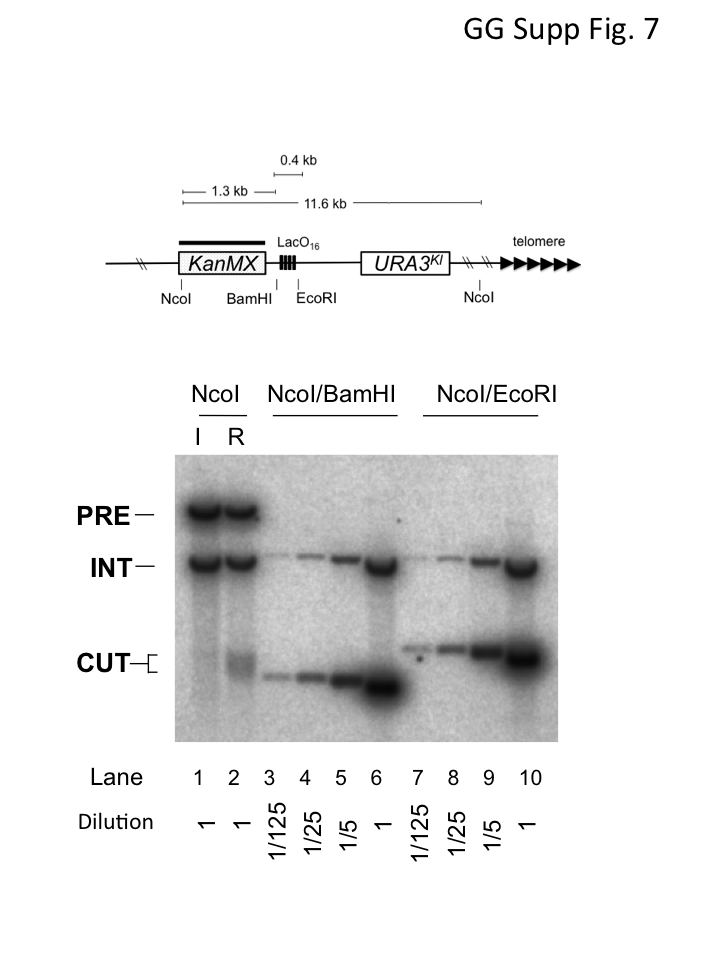

Supplement: S7 Fig — Cells containing the LacO16-URA3 cassette were transformed with pGAL-LacI-RAP1 (R) or pGAL-LacI (I). Transformants were initially grown in 2% sucrose and then incubated with 2% galactose and 0.5% glucose for 4 hr. Genomic DNA from cells expressing LacI or LacI-Rap1 was digested with NcoI (Lane 1–2). The LacO16 repeat is cloned between the BamHI and EcoRI sites (see also Fig 4A). Genomic DNA from the control cells carrying the control vector was digested with either NcoI and BamHI or NcoI and EcoRI, and serially diluted (Lane 3–10). Digested DNA was analyzed by Southern blot as in Fig 4B. It was estimated that 3% of cells received DNA breaks at the LacO16 locus after LacI-Rap1 expression by using a Typhoon imaging system. (TIFF) [file pgen.1005283.s007.tiff]

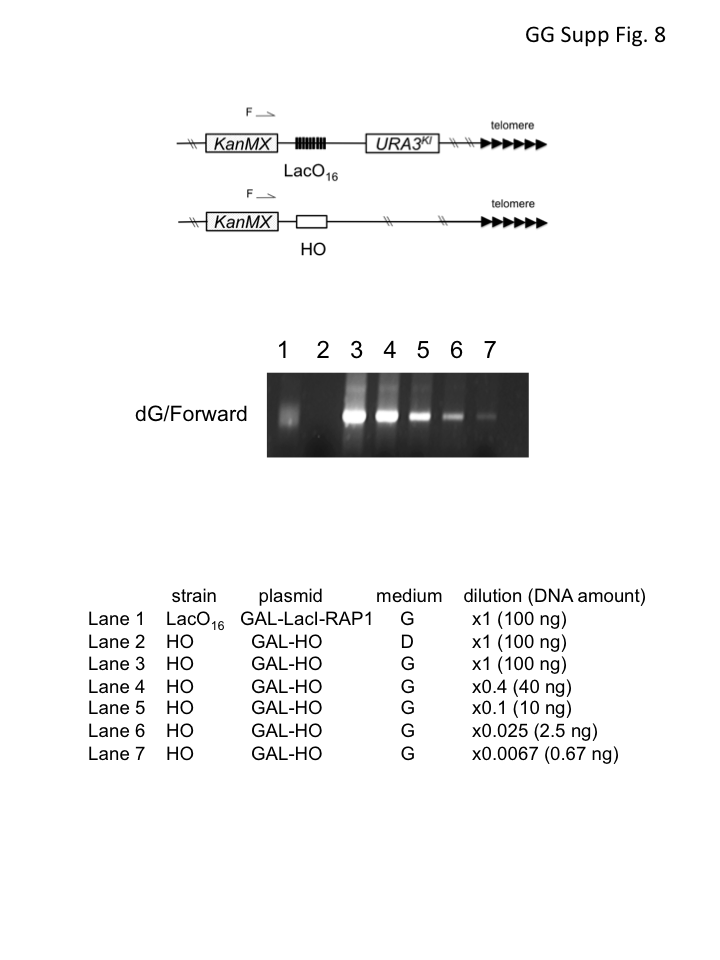

Supplement: S8 Fig — LacO16-URA3 cells carrying pGAL-LacI-RAP1 were treated as in Fig 4C (Lane 1). HO cells carrying pGAL-HO were precultured in sucrose and then incubated with 2% glucose (D) to repress HO expression (Lane 2) or 2% galactose (G) to induce HO expression (Lane 3–7) for 4 hr. Genomic DNA was extracted and examined by the TdT-PCR assay as in Fig 4C. Samples from HO expressing cells were serially diluted before PCR (Lane 3–7). 80% of HO cells were found to induce a DNA break at the HO recognition site after HO induction [78]. It was estimated that 3% of LacO16-URA3 cells received DNA breaks with the LacO16 repeat sequence after LacI-Rap1 expression. (TIFF) [file pgen.1005283.s008.tiff]

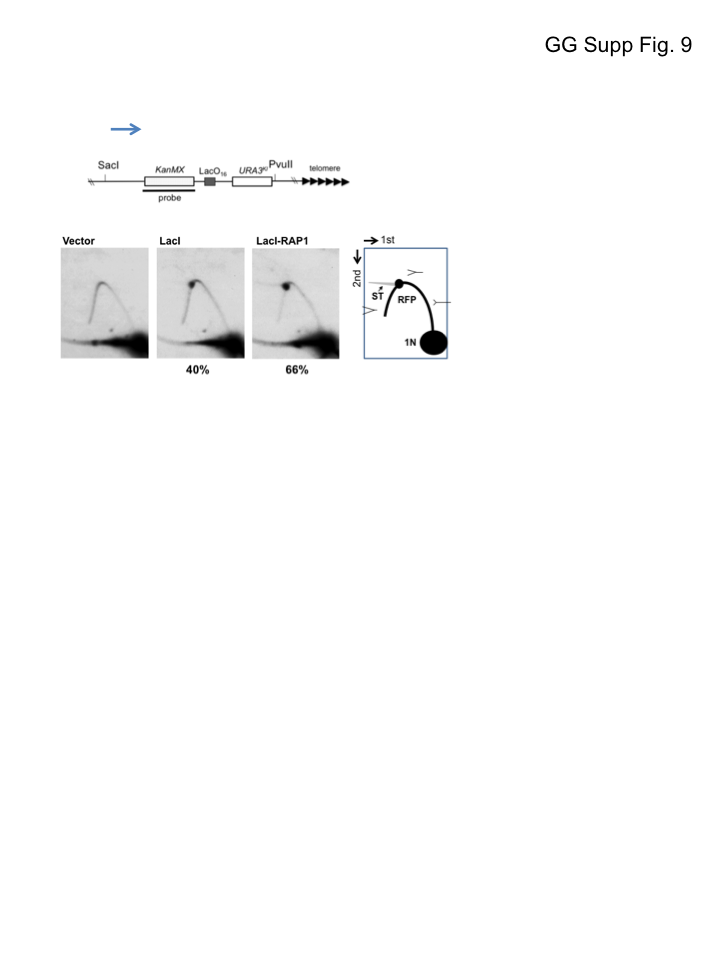

Supplement: S9 Fig — CsCl gradient purified DNA was digested with SacI and PvuII and analyzed by two-dimensional gel electrophoresis using the indicated probe. The probe detects a 5.5 kb SacI-PvuII fragment. The LacO16 repeat is located 3.2 kb from the SacI site and 1.9 kb from the PvuII site. RFP represents replication fork pausing. Note that some parts of RFP signal are smearing (ST). The number (%) below each panel denotes the ratio of the signal of RFP to that of total replication intermediates. The arrow indicates the direction of replication fork movement. (TIFF) [file pgen.1005283.s009.tiff]

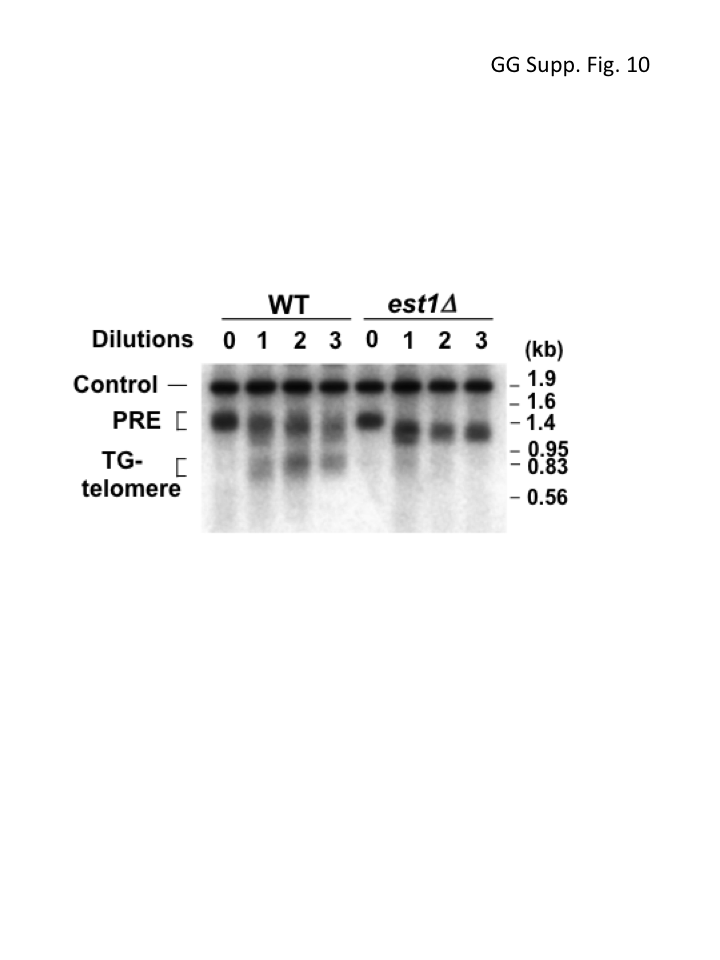

Supplement: S10 Fig — TG33-LacO16-tel cells and TG33-LacO16-tel est1Δ cells carrying the URA3-marked EST1 plasmid were transformed with pGAL-LacI-RAP1. Transformants were treated and examined as in Fig 7E. (TIFF) [file pgen.1005283.s010.tiff]
