# Supplementary material for: Binding of Multiple Rap1 Proteins Stimulates Chromosome Breakage Induction during DNA Replication
Source: PLoS Genet. 2015 Aug 11;11(8):e1005283. doi: 10.1371/journal.pgen.1005283 (PMC4532487; doi:10.1371/journal.pgen.1005283)
Supplement: S1 Table — All the strains are isogenic and the detailed construction is described in Materials and Methods. (DOCX) [file pgen.1005283.s011.docx]

Support information Table 1. List of strains used in this study.

__________________________________________________________________________________________

KSC3064 *ADH4::KanMX-URA3*

KSC3065 *ADH4::KanMX-TG_81_-URA3*

KSC3066 *ADH4::KanMX-TG_250_-URA3*

KSC3067 *ADH4::KanMX-TG_250_-URA3 rad52∆::ura3*

KSC3096 *ADH4::KanMX-TG_250_-URA3 rap1-∆C::LEU2*

KSC3098 *ADH4::KanMX-TG_250_-URA3 rap1-∆N::NatMX*

KSC3105 *ADH4::KanMX-LacO_16_-URA3*

KSC3626 *ADH4::KanMX-LacO_16_-URA3* *rad52∆::LEU2 yku70∆::HphMX*

KSC3357 *ADH4::KanMX-LacO_16_-URA3* *cdc17-1*

KSC3337 *ADH4::KanMX-LacO_16_-URA3* *sml1∆::LEU2*

KSC3361 *ADH4::KanMX-LacO_16_-URA3* *mec1∆::LEU2 sml1∆::LEU2*

KSC3627 *ADH4::KanMX-LacO_16_-URA3* *mrc1∆::LEU2*

KSC3628 *ADH4::KanMX-LacO_16_-URA3* *tof1∆::LEU2*

KSC2217 *ADH4::KanMX-HO*

KSC2567 *ADH4::KanMX-TG_81_-HO*

KSC3262 *ADH4::KanMX-TG_250_-URA3 ACE1-UBR1::ura3 ACE1-ROX1::ura3 ANB1-rap1-(∆)::KanMX*

KSC3263 *ADH4::KanMX-TG_250_-URA3 ACE1-UBR1::ura3 ACE1-ROX1::ura3*

KSC3279 *ADH4::KanMX-*telomere (TG-telomere)

KSC3280 *ADH4::KanMX-LacO_4_*-telomere

KSC3282 *ADH4::KanMX-TG_33_-LacO_16_*-telomere

KSC3283 *ADH4::KanMX-TG_33_-LacO_16_-telomere est1∆::ura3* [YCpU-EST1]

KSC3284 *ADH4::KanMX-TG_33_-LacO_16_-telomere rad52∆::URA3*

KSC3501  *ADH4::KanMX-ura3-∆C YER186::HphMX-ura3-∆N*

KSC3502 *ADH4::KanMX-ura3-∆C-LacO_4_ YER186::HphMX-ura3-∆N*

KSC3503 *ADH4::KanMX-ura3-∆C-LacO_8_ YER186::HphMX-ura3-∆N*

KSC3504 *ADH4::KanMX-ura3-∆C-LacO_16_ YER186::HphMX-ura3-∆N*

KSC3505 *ADH4::KanMX-ura3-∆C-LacO_16_ YER186::HphMX-ura3-∆N rif1∆::ura3 rif2∆::HphMX*

KSC3506 *ADH4::KanMX-ura3-∆C-LacO_16_ YER186::HphMX-ura3-∆N sir3∆::NatMX sir4∆::LEU2*

KSC3507 *ADH4::KanMX-ura3-∆C-LacO_16_ YER186::HphMX-ura3-∆N sml1∆::LEU2*

KSC3508 *ADH4::KanMX-ura3-∆C-LacO_16_ YER186::HphMX-ura3-∆N mec1∆::LEU2 sml1∆::LEU2*

KSC3596 *rap1∆::NatMX* [YCpU-RAP1]

KSC3614 *ADH4::KanMX-ura3-∆C-TetO_8_ YER186::HphMX-ura3-∆N*

_________________________________________________________________________________________

All the strains are isogenic to KSC1508 (*MATa-inc*, *ade1, his2, trp1, ura3, leu2*).
