# Supplementary material for: Binding of Multiple Rap1 Proteins Stimulates Chromosome Breakage Induction during DNA Replication
Source: PLoS Genet. 2015 Aug 11;11(8):e1005283. doi: 10.1371/journal.pgen.1005283 (PMC4532487; doi:10.1371/journal.pgen.1005283)
Supplement: S2 Table — (DOCX) [file pgen.1005283.s012.docx]

Support information Table 2. List of oligonucleotides used in this study

Name Sequence

KSX001 5'-AGCGGATAACAATTTCACACAGGA-3'

KSX002 5’-CGCCAGGGTTTTCCCAGTCACGAC-3’

KSX006 5'-AATTAACCCTCACTAAAGGGAAC-3'

KSX007 5'-TAATACGACTCACTATAGGGCGA-3'

KSX050 5'-TCCGACTCGTCCAACATC-3'

KSX089 5'-GACTCACGTTTCGAGGCCGCG-3'

KS1120 5'-CTCGGATCCGTTGATTGTATGCTTGGTATAGC-3'

KS1871 5'-AAAGAATTCCCCCTGCACCCACACACTCTCTCACATC-3'

KS1868 5'-AAAGAATTCCTGAGTGCATTTGCAACATG-3'

KS1876 5'-AAAGAGCTCTTCATCAACCTCCACAGAAAG-3'

KS2043 5'-AAACTCGAGTTTATTGTGGGAGGGCAAAGCG-3'

KS2045 5'-AAACCGCGGATGCTCGAGAAGAGGGCAAGAAATTATAGTTCTC-3'

KS2130 5'-AAAGGATCCATGTCCACAAAATCATATACCAGTAG -3'

KS2131 5'-AAATCTAGAAGATCTTTAATGGGGAGCGCTGATTC -3'

KS2181 5'-AAACTCGAGCCTGAGTGCATTTGCAACATG-3'

KS2183 5'-AAAAAACAATTGAGATCTGGGTGTGGTGTGGG TGTGGTGTGGGTGTGGTGGCAGG-3'

KS2184 5'-AAACTCGAGCGTTCGTTCGACTGATGAGC-3'

KS2186 5'-ATTTAAACTTATCCGGAACATCCTTCCTGTCTTC GTTAGCGTAGATTTCTCAGGAAACAGCTATGACC-3'

KS2187 5'-ATGCTGTGGCATCCTGATGGTTACGAGCCTCG

AGTAAAGGCTATAGAAGAGTTGTAAAACGACGGCCAG-3'

KS2799 5'-AAACCGCGGGAATCGCATTCTAATGGTGTAGA-3'

KS2812 5'-CTCACGCGTTCGTCTCATCTATAATAGAAATAT-3'

KS2994 5'-AAACTCGAGAGAGTTGGAAGCAGCGGCAG-3'

KS2995 5'- AAACTCGAG CCAAAAATTGCAACAACAGATCT-3'

KS2996 5'- AAACTCGAG AGACTAGAGTACGTTTATGAGG-3'

KS2997 5'- AAA CTC GAG TTTCCTTTTAATTGATGGTGGCAA-3'

KS3004 5'-GTCGGCAATGTCCCAAGTGG-3'

KS3005 5'-CGCGGCCTCGAAACGTGAGTCGGGTTCTACCCACTACTAG-3'

KS3260 5'-GACAAAGGTGGCGGATCCTTGCTGATTGGCGTTGCCTCCTCCAG-3'

KS3261 5'-GCCAATCAGCAAGGATCCGCCACCTTTGTCCGCCAGTTGTTGTGC-3'

Poly(dG) 5’-AAATCTAGAGGGGGGGGGGGGGGGGGG-3’

TdT forward 5’-GCATAAGCTTTTGCCATTCTCAC-3’

TdT reverse 5'-CTCGGATCCGTTGATTGTATGCTTGGTATAGC-3'

SIR3 disruption F

5’-CACATAAGCAGCCCTTTCATCACCTTCCTTACAGGGGTTTAAGAAAGTTGCAGG

AACAGCTATGACC-3’

SIR3 disruption R

5’-ATCTATGGCGGAAGTGAAAATGAATGTTGGTGGTCAAATGCAGTCCA

TATGTTGTAAAACGACGGCCAG-3’

SMC2 forward 5'-AAACTCGAGCAAGAAACCAACTGCAGGATC -3'

SMC2 reverse 5'-AAAGAATTCGCAACAGTATAAGAATTGATCCTAC -3'

____________________________________________________________________________
