# Supplementary material for: Binding of Multiple Rap1 Proteins Stimulates Chromosome Breakage Induction during DNA Replication
Source: PLoS Genet. 2015 Aug 11;11(8):e1005283. doi: 10.1371/journal.pgen.1005283 (PMC4532487; doi:10.1371/journal.pgen.1005283)
Supplement: S1 Text — Details of plasmid construction and plasmid information are described. (DOCX) [file pgen.1005283.s013.docx]

**Supplemental information S1 Text**

**Plasmid construction**

**pT81-URA3**

To construct the pT81-URA3 plasmid, we first prepared an *ADH1* fusion to *the K. lactis URA3* gene. The *ADH1* promoter was amplified from budding yeast genomic DNA with the primer pair (KS1120/1868) and cleaved with BamHI and EcoRI. The *K. lactis URA3* was amplified by PCR using pWJ1076 [[1](#_ENREF_1)] as a template with the primer pair (KS2130/2131) and digested with BamHI and XbaI. The resulting two fragments were cloned into EcoRI-XbaI treated YCplac22 [[2](#_ENREF_2)], generating YCp22A-URA3. The BglII-EcoRI fragment containing the *ADH1-URA3* fusion was cloned into BamHI-EcoRI-treated pTG_81_-HO [[3](#_ENREF_3)], generating pT81-URA3.

**pT250-URA3**

The pT250-URA3 plasmid was constructed as follows. A DNA fragment containing TG_250_ was amplified from the ADE2-TG_250_ plasmid [[4](#_ENREF_4)] with the primer pair (KS2082/2083). The resulting PCR fragment was digested with EcoRI and XhoI, and cloned into EcoRI/XhoI-treated pT81-URA3, generating pT250-URA3.

**pNO-URA3**

To construct the pNO-URA3 plasmid, an *ADH1-URA3* fragment was amplified from YCp22A-URA3 with the prime pair (KS2131/2181). The resulting fragment was digested with BglII and XhoI, and cloned into BamHI-XhoI-treated pTG_81_-HO, removing the 81 bp TG sequence and introducing the URA3 fragment.

**pO16-URA3**

To construct the pO16-URA3 plasmid, we first duplicated the lacO_8_ sequence on placO_8_-HO [[5](#_ENREF_5)], generating the placO16-HO plasmid. The EcoRI-XhoI fragment containing the lacO_16_ repeat replaced the EcoRI-XhoI fragment of pT81-URA3, resulting in pO16-URA3.

**pO4-TG81-HO**

An MfeI-EcoRI fragment containing the LacO_4_ sequence from the placO4-HO plasmid [[5](#_ENREF_5)] was cloned into MfeI-EcoRI digested pTG_81_-HO, generating pO4-TG81-HO. The LacO4 repeat contains four natural lactose operators (LacI-binding sites) from the lactose operon of *E. coli*. The lactose operon has one primary operator and two ancillary operators [[6](#_ENREF_6)]. We combined these three operator sequences, generating the LacO_4_ repeat [[5](#_ENREF_5)]. The LacO_8_ and LacO_16_ repeat sequences were generated from the LacO_4_ repeat.

**pTG33-O16-TG81-HO**

The pTG33-O16-TG81-HO plasmid was constructed in two steps. First, we prepared the placO16-TG81-HO plasmid containing the LacO_16_ repeat from placO8-TG-HO by fusing the lacO_8_ sequence in tandem as described [[5](#_ENREF_5)]. Second, a DNA fragment containing both the KanMX marker and a 33 bp TG sequence was amplified from pTG_81_-HO using the primer pair (KSX006/KS1781). The resulting KanMX-TG33 fragment was digested with EcoRI and SalI, and cloned into MfeI-SalI-treated placO16-TG81-HO plasmid, generating pTG33-O16-TG81-HO plasmid.

**pUN-O0, pUN-O4, pUN-O8, pUN-O16 or pUN-tetO8 plasmid**

The pUN-O0, pUN-O4, pUN-O8 or pUN-O16 plasmid was prepared as follows. A DNA fragment distal to the *ADH4* locus (*ADH4*-distal fragment) was amplified by PCR from the pSD155 plasmid [[7](#_ENREF_7)] with the primer pair (KSX007/KS2183), and digested with MfeI and NotI. An *URA3* fragment lacking the C-terminal region (ura3-ΔC) was amplified from YCp22A-URA3 with the primer pair (KS2181/2812) and digested with MfeI and XhoI. These fragments were cloned into NotI-XhoI treated pHO [[3](#_ENREF_3)], generating pUN-O0. The MfeI-XhoI ura3-ΔC fragment and the MfeI-BglII lacO repeat fragment from placO4-HO, placO8-HO and placO16-HO were cloned into XhoI-BamHI-treated pHO, generating pUN-O4, pUN-O8 and pUN-O16, respectively. The MfeI-XhoI ura3-ΔC fragment and the MfeI-BglII tetO repeat fragment from ptetO8-HO [[3](#_ENREF_3)] were cloned into XhoI-BamHI-treated pHO, generating pUN-tetO8.

**pUC18 containing the ura3-**Δ**N-Hph cassette**

An URA3 fragment lacking the N-terminal region (ura3-ΔN) was amplified from YCp22A-URA3 with the primer pair (KSX001/KS2184), and digested with HindIII and XhoI. The HindIII-XhoI ura3-ΔN fragment and a SacI-SalI fragment containing HphMX from pAG32 [[8](#_ENREF_8)] were cloned into pUC18, generating pUC18-ura3-ΔN-Hph. The ura3-ΔN-Hph cassette was amplified with the primer pair (KS2186/2187), and introduced at the *YER186* locus on chromosome V.

**pGAL-LacI-RAP1, pGAL-LacI**-RAP1, pGAL-LacI-GAL4, pGAL-LacI-RAP1 (224-663) and pGAL-LacI**

The plasmids containing LacI or LacI-fusion genes were constructed as follows. The SacI-SalI DNA fragment containing the lacI coding sequence [[5](#_ENREF_5)] was transferred from placI to YCpG22, resulting in pGAL-LacI. YCpG22 is a YCplac22 plasmid containing the *GAL1* promoter [[9](#_ENREF_9)]. The coding sequence of *RAP1* from ptetR-RAP1 was cloned into SacI/SacII-treated pGAL-LacI, generating pGAL-LacI-RAP1. The expression of LacI-Rap1 complements *rap1* deletion mutations as found for that of TetR-Rap1 [[3](#_ENREF_3)]. The pGAL-LacI**-RAP1 plasmid was constructed using two PCR fragments as follows. The first DNA fragment was amplified by PCR with the primer pair (KSX002/3261) and digested with EcoRI and BamHI. The second DNA fragment was amplified by PCR with the primer pair (KS3260/2043) and digested with BamHI and SacII. The LacI** mutation generates a BamHI restriction site. These two fragments were cloned into EcoRI-SacII-treated pGAL-LacI-RAP1. The *GAL4* coding sequence was amplified with the primer pair (KS2165/2166) and cloned into SacI/SacII-treated pGAL-LacI, creating pGAL-LacI-GAL4. A DNA fragment containing the Rap1 middle domain (224-663) was amplified by PCR with the primer pair (KS2799/1876) and similarly cloned into pGAL-LacI, generating pGAL-LacI-RAP1 (224-663).

**Deletion constructs of the Rap1 central region in pGAL-LacI-RAP1**

DNA fragments were amplified with the following primer pairs using pGAL-LacI-Rap1. Amplified DNA fragments were digested with XhoI and self-ligated. Primer pair: KS2043/2996 for ΔM1, KS2997/2045 for ΔM2, KS2043/2045 for ΔM1-M2, KS3059/2995 for ΔTA and KS2043/2995 for ΔM1-M2-TA.

**Other plasmids**

YEp-RAP1 or YCp-RAP1 is a *TRP1*-marked YEplac112 high copy plasmid or pRS316 carrying the *RAP1* gene, respectively. The *RAP1* gene, amplified by the primer pair (KS1876/2030), was digested with EcoRI and SacI, and cloned into either YEplac112 [[2](#_ENREF_2)] or pRS316 [[10](#_ENREF_10)]. The pTG81-HO, ptetO8-HO or ptetR-RAP1 plasmid has been described [[3](#_ENREF_3)]. YCpT-TetR and YCpT-TetR-Rap1 are a *TRP1*-marked YCplac22 version of ptetR and ptetR-RAP1, respectively [[3](#_ENREF_3)]. The placO4-HO, placO8-HO, placO8-TG-HO, pGAL-HO or YCpU-EST1 plasmid was described [[5](#_ENREF_5)]. The oligonucleotides used in this study are listed in Support information Table 2.

**References**

1. Reid RJ, Lisby M, Rothstein R (2002) Cloning-free genome alterations in Saccharomyces cerevisiae using adaptamer-mediated PCR. Methods Enzymol 350: 258-277.

2. Gietz RD, Sugino A (1988) New yeast-*Escherichia coli* shuttle vectors constructed with in vitro mutagenized yeast genes lacking six-base pair restriction sites. Gene 74: 527-534.

3. Hirano Y, Fukunaga K, Sugimoto K (2009) Rif1 and Rif2 inhibit localization of Tel1 to DNA ends. Mol Cell 33: 312-322.

4. Negrini S, Ribaud V, Bianchi A, Shore D (2007) DNA breaks are masked by multiple Rap1 binding in yeast: implications for telomere capping and telomerase regulation. Genes Dev 21: 292-302.

5. Fukunaga K, Hirano Y, Sugimoto K (2012) Subtelomere-binding protein Tbf1 and telomere-binding protein Rap1 collaborate to inhibit localization of the Mre11 complex to DNA ends in budding yeast. Mol Biol Cell 23: 347-359.

6. Lewis M (2005) The lac repressor. C R Biol 328: 521-548.

7. Diede SJ, Gottschling DE (1999) Telomerase-mediated telomere addition in vivo requires DNA primase and DNA polymerases alpha and delta. Cell 99: 723-733.

8. Goldstein AL, McCusker JH (1999) Three new dominant drug resistance cassettes for gene disruption in Saccharomyces cerevisiae. Yeast 15: 1541-1553.

9. Kondo T, Matsumoto K, Sugimoto K (1999) Role of a complex containing Rad17, Mec3, and Ddc1 in the yeast DNA damage checkpoint pathway. Mol Cell Biol 19: 1136-1143.

10. Sikorski RS, Hieter P (1989) A system of shuttle vectors and yeast host strains designed for efficient manipulation of DNA in Saccharomyces cerevisiae. Genetics 122: 19-27.
